# The "face race lightness illusion": An effect of the eyes and pupils?

**DOI:** 10.1371/journal.pone.0201603

**Published:** 2018-08-02

**Authors:** Bruno Laeng, Kenneth Gitiye Kiambarua, Thomas Hagen, Agata Bochynska, Jamie Lubell, Hikaru Suzuki, Matia Okubo

**Affiliations:** 1 Department of Psychology, University of Oslo, Oslo, Norway; 2 Kenya Methodist University, Meru, Kenya; 3 Department of Language and Literature, Norwegian University of Science and Technology, Trondheim, Norway; 4 Helen Wills Neuroscience Institute, University of California at Berkeley, Berkeley, United States of America; 5 Department of Psychology, Senshu University, Tokyo, Japan; RMIT University, AUSTRALIA

## Abstract

In an internet-based, forced-choice, test of the ‘face race lightness illusion’, the majority of respondents, regardless of their ethnicity, reported perceiving the African face as darker in skin tone than the European face, despite the mean luminance, contrast and numbers of pixels of the images were identical. In the laboratory, using eye tracking, it was found that eye fixations were distributed differently on the African face and European face, so that gaze dwelled relatively longer onto the locally brighter regions of the African face and, in turn, mean pupil diameters were smaller than for the European face. There was no relationship between pupils’ size and implicit social attitude (IAT) scores. In another experiment, the faces were presented either tachistoscopically (140 ms) or longer (2500 ms) so that, when gaze was prevented from looking directly at the faces in the former condition, the tendency to report the African face as “dark” disappeared, but it was present when gaze was free to move for just a few seconds. We conclude that the presence of the illusion depends on oculomotor behavior and we also propose a novel account based on a predictive strategy of sensory acquisition. Specifically, by differentially directing gaze towards to facial regions that are locally different in luminance, the resulting changes in retinal illuminance yield respectively darker or brighter percepts while attending to each face, hence minimizing the mismatch between visual input and the learned perceptual prototypes of ethnic categories.

## Introduction

**Fig 1:**
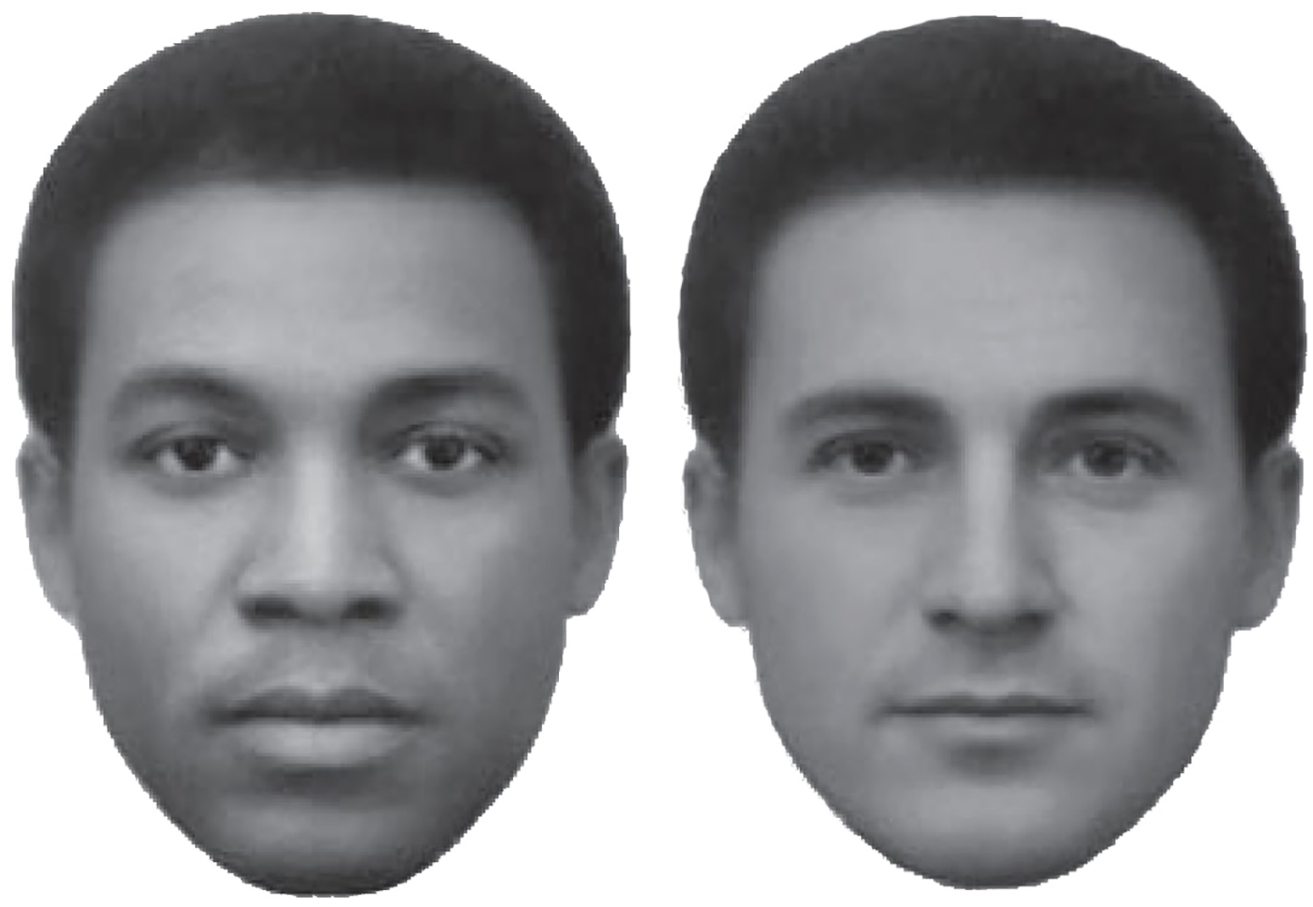
The African face (left) and the European face (right) have the same average luminance and are closely matched in size (e.g., in Experiment 2: mean pixels’ brightness or L_mean_ = 112.5, *SD* = 41, in HSL/RGB coordinates). Because the perceived difference in skin tone persists even after the observer is informed that the two face images deliver the same average intensity of light to the eyes, such a visual demonstration has been included in the “library” of optical illusions and it is often referred to as the *Face Race Illusion* (source: “Distortions in the perceived lightness of faces: the role of race categories,” by Daniel T. Levin and Mahzarin R. Banaji, 2006, *Journal of Experimental Psychology*: *General*, vol. 1358:4).

The so-called “face race lightness illusion” has been considered since its discovery [1] as a compelling example of the effect of (social) world knowledge onto perception or, in psychological terms, of “top-down” cognitive effects on visual perception. Differently from other putative evidence for the effects of knowledge or beliefs on perception, and similarly to classic optical illusions, it can be easily demonstrated by simply looking at the two faces in Fig 1, shown side-by-side as gray-level photo images. The faces are easily categorized by observers as respectively of African and European ethnicities since recognition of ethnicity is strongly based on differences in face features or morphology [2, 3]. What gives this particular pair of face images the status of an illusion is that observers experience the faces as having different skin-tone lightness despite the two facial images have the same average luminance.

Specifically, the fact that in this demonstration the African face is seen generally darker in skin tone than the European face would seem to be consistent with shared prior knowledge of the typical skin tones of the corresponding ethnicities [1], yet this visual experience would seem in conflict with the average physical light energy that actually stimulates the eye. The proposed influence of social knowledge on perception can be attributed to a general tendency of human minds to simplify the process of categorization by automatically recalling schematic perceptual information (i.e., by relying on ‘categorical thinking’; [4]). Although this process when applied to other persons tends to stereotype them, since it does not consider them in terms of their unique constellations of attributes, the attribution of social categories (like ethnicity, gender, age, etc.) allows accessing a wealth of categorically-related information from long-term memory [5] that can be useful when relating to other (often unknown) individuals. The learned social categories would also include information about the typical perceptual features for the accessed social category (e.g., the average face shape, typical skin tone, etc. [6–8]), possibly according to beliefs in the diagnosticity of the features [9]. Among these visual cues, skin tone is a highly salient visible attribute that can apparently already shape social categorization in early childhood [10].

The construction of social categories would thus seem to reflect a general and automatic mechanism of the cognitive system and it should not be considered per se a symptom of a prejudiced stance towards out-group individuals. Nevertheless, it is important to stress in this context that the pervasive social importance of skin color in human affairs only indicates an exaggerated perception and sensitivity within societies to just one obvious attribute of human appearance [11] which is however insignificant for biological taxonomy. That is, skin tone does not reflect an actual biological difference that can be used to parse the human species into sub-species, given that genetic variation is preponderantly accounted for by differences between individuals within populations and not their membership in a specific ‘race’ or ethnic group [12]. In fact, according to the anthropologist Jonathan Marks (p. 69, [13]), the very idea of race “turns out to be an optical illusion.”

### The present study

It seems likely that the face race lightness illusion is ubiquitous and experienced similarly by people of different ethnicities and cultures. However, there exists as of yet no systematic investigation about the pervasiveness of the face-race lightness illusion and whether it can be generalized to different populations. The original study by Levin and Banaji [1] included some African American participants, but in small proportions for allowing meaningful comparisons with the European-descendent majority of participants. Because the classic account of this illusion posits that it depends on knowledge of the typical physical features of the two ethnicities represented in the images, it is reasonable to hypothesize that the “strength” of the illusion may vary with individuals’ geographical location and consequently to their direct social exposure to individuals with darker or lighter skin tones.

In fact, most psychological studies are conducted in so-called WEIRD societies (i.e., “Western Educated Industrialized Rich Democratic”; see [14]). This can be a limiting factor when generalizing findings and in relation to claims of universality of specific psychological effects, including the susceptibility to optical illusions (e.g., the Müller-Lyer illusion; [15]). Since illusions can be sensitive to the perceptual context [16, 17], one cannot exclude variability to the face-race lightness illusion based on different exposure, especially in geographical locations where one or both of the ethnicities are particularly rare. Hence, the initial goal of the present study is to assess the presence and strength of the face-race lightness illusion with respondents widely differing in nationality and ethnicity, where the level of exposure to individuals of either African or European ethnicity is either in favor of the former or of the latter ethnicity.

**Fig 2:**
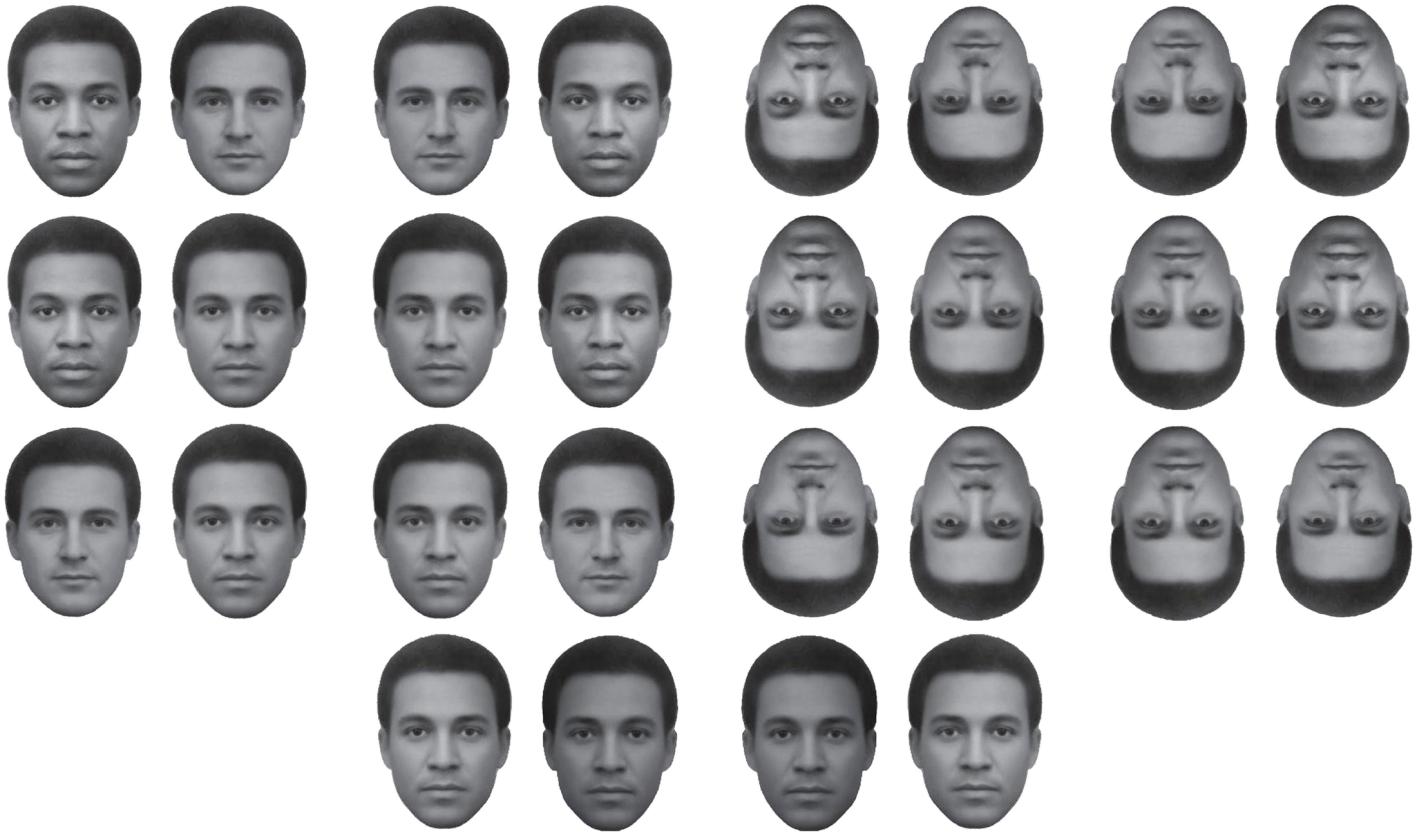
Stimuli used in the crowdsourcing, internet-based, Experiment 1. The first, from top, row corresponds to the standard stimuli (L_mean_ = 112.7, *SD* = 41), one version with African face on the left or right side of the pair on the right side there are the same stimuli rotated 180° or ‘upside-down’ faces. The second and third rows show the European face paired with a 50% face morph of the European and African face and the African face paired with the same 50% face morph, plus the inverted stimuli. All face stimuli were equiluminant except the face pairs in the fourth bottom row which shows “catch trials”, with the face morph presented twice (as the first and last trial) but one version being visibly darker than the other stimulus (L_mean_ = 91.8, *SD* = 32).

According to the ‘contact hypothesis’ [7], during an individual’s development, when the family is one’s main social input, the structure of so-called ‘face-space’ becomes complete and in a way “crystallizes.” Later on, one may begin to encounter a sufficient number of individuals of other ethnicities, but the acquired face-space structure encodes with difficulty the variations in physical dimensions of these other faces that tend to become tightly clustered, according to the ‘face-space model’, in a demarked region of face-space [18]. This learning difficulty possibly leads to another social bias affecting the perception of one’s own ethnicity as consisting of a wider variety of skin colors and tones than those of other ethnicities (i.e., the “they are all alike” illusion [19]). Indeed, as the “other-race bias” in memory for faces testifies, recognition performance of ethnicities that are uncommon for a group of participants can be significantly lower than for faces of the “own race” (e.g., [20–22]). For example, we assume that respondents from East African countries (e.g., Kenya) have preponderantly been exposed to ethnically African individuals, especially compared to respondents from European countries (e.g., Norway or Poland) that will instead tend to have preponderant exposure to European individuals. In contrast, in East Asian countries (e.g., Japan), both of the above ethnicities can be encountered quite rarely. Hence, the first experiment assessed the presence and strength of the bias, by collecting responses about the relative lightness of skin tone of images like the one in Figs 1 and 2. This was accomplished by recruiting a largely diverse sample of participants by use of internet-based, crowdsourcing, sampling methods [23].

The other central goal of the present study is to provide a principled account of the perceptual and/or cognitive mechanisms underlying the presence and strength of the illusion. In particular, we attempt here to throw light on this ‘dark’ bias (no pun intended) for the African face by revealing attentional mechanisms that can generate the illusory effect. In our opinion, there are four plausible accounts for the face race lightness (FRL) illusion or perceptual ‘bias’:

1. The ‘Prototype-based Illusion’ account: the FRL bias is a perceptual distortion of the visual input resulting from influence of (social) world knowledge on the percept and therefore an exemplary case of top-down cognitive effects on visual perception. In other words, the sensory information conflicts with one’s perceptual memory (i.e., the remembered face prototype of one ethnicity may look darker than another); hence, an internal transformation of the sensory information generates a new percept with sensible features that are consistent with the statistical information gleaned from past perceptual experience. This account is the one proposed in the original study by Levin & Banaji [1] and consistent with empirical evidence in luminance-matching experiments.
2. The ‘Stereotype-based Illusion’ account: the FRL bias is a perceptual distortion of the visual input resulting from (social) stereotypes or prejudice towards specific ethnicities, so that one sees a luminance difference where there is not any (e.g., [24]). Indeed, skin color is the most obvious visible attribute of the human body and it has been historically the primary characteristic used to classify people into distinct geographic groups or ethnicities and even into purportedly genetically distinct groups [11, 12]. More specifically, stimuli are judged as brighter when they also tend to be the socially preferred one (e.g., [25, 26]). Although Levin & Banaji [1] found no effect of explicit race prejudice on the strength of the face-race lightness illusion in their American participants, it remains possible that implicit forms of prejudice could play a role in the occurrence of the bias and its strength.
3. The ‘Artifact’ account: the so-called FRL illusion is not due to a perceptual transformation and the bias is not necessarily dependent on the attribution of specific ethnicities to the two stimuli. The phenomenon is instead an artifact elicited by uncontrolled but salient visual properties within the images used in its demonstration. Firestone and Scholl [27, 28] introduced this account, as a case study within a more general discussion about “cognitive penetrability” in perception or top-down cognitive biases. By showing degraded (blurred) versions of the original images, they found that participants continued to yield the same bias, despite being unable to categorize the stimuli as either ethnicity (but see [29] for a rebuttal). Most interestingly, Firestone and Scholl hinted at the possibility that spontaneous patterns of eye movements on such salient features could be at the basis of the response bias and explicitly stated that a face could look darker if gaze or attention was captured by its salient dark parts. In fact, Levin & Banaji had already entertained and rejected such an account by finding that the bias was present, albeit greatly weakened, in luminance-matching task with simple line drawings consisting of only the face contours and no grey-tones. However, eye fixations were not analyzed in any of these experiments. Finally, Firestone and Scholl [28] mentioned “task demands” effects [30] as possibly playing a role when using the luminance-adjustment method. Nevertheless, they described the FRL effect as compelling, as it is the case for many classic optical illusions.
4. The ‘Predictive sampling’ account: the FRL bias is the result of selective sampling of the visual input that is driven by (social) world knowledge, so that the most consistent level of luminance present in the visual input would be searched for when looking at a face of a specific ethnicity, so as to “confirm” prior expectations. This account is similar to the first two accounts, since a categorization process relying on lifelong statistical regularities experienced in one’s visual environment would generate and construct perceptual predictions to facilitate perception, in this case related to the ethnicities. However, in the present account, the illusion would not necessarily emerge as the expression of a post-sensory, distortion or postdiction, mechanism but as a prediction. Differently from the artifact-based account, the present account is consistent with a “weak” form of top-down cognitive influence on sensory sampling of each face. In this view, the observer would literally ‘see’ one face as either less or more bright than the other. Crucially, selective gaze patterns will bring about systematic differences in levels of retinal illuminance when attending to one face or the other. This last account is the one favored in the present study and we present here empirical evidence in its support.

## Experiment 1

All of the above accounts would seem to have the ability to generate specific predictions about the presence and strength of the FRL in individuals belonging to different ethnicities. The ‘stereotype-based’ account would predict that the illusory bias and its strength vary in relation to the degree of the negative/positive attitude towards each ethnicity, and this could reflect the observers’ racial prejudice either explicitly or only implicitly. According to the ‘prototype-based’ account, even unprejudiced observers, with limited exposure to one of the target ethnicities, would rely on prototypical knowledge of physical characteristics of an ethnicity to a greater extent than observers who have more experience with other-race individuals. In the latter case, the knowledge of the prototype would be more precise or possibly allow a broader range and variability of expected skin-tones. Hence, in the light of the above accounts, one could expect that the geographical location of respondents may modulate the prevalence of the reported illusory effect. The ‘predictive sampling’ account would make similar predictions to those of the prototype-based account, since the strategic sampling of the visual input would also reflect the corresponding perceptual prototypes. Within this account, prejudice may also play a role in the strategic deployment of visual sampling of the stimuli. Finally, the ‘artifact-based’ account posits that the effect is not dependent on identifying the ethnicity of the faces. Hence, one would not expect differences in the prevalence or strength of the effect across individuals of different ethnicities or social attitudes, as long as the experiment employs the same stimuli used in the standard FRL demonstrations. Moreover, it should not matter whether a) the faces are presented upside-down thereby making it difficult to recognize ethnicity or facial information [31]; or b) if the difference in the faces’ ethnicities is made less salient [1, 32], by use of facial morphs of ambiguous ethnicity. Hence, in the present experiment we also paired in some trials, either upside-down faces or face pairs where one of the standard stimuli was a morph of the African and European face stimuli. We expected that these ambiguous stimuli would yield a weaker FRL bias than the standard pair of upright faces, since with inversion the face perception mechanisms are inefficient in processing a face configural structure [33], which in turn should affect the ability to identify a person’s ethnicity.

### Methods

#### Participants

We recruited all participants via crowdsourcing on the web. All participants consented to the inclusion of their responses in a study and remained fully anonymous and not identifiable. There were several methods of recruitment, one based on paid membership to crowdsourcing platforms (*Crowdflower* and *Prolific Academic*) and the other by free participation on an alternative web platform. In the latter case, we obtained recruitment via several announcements to colleagues in various universities in Italy, Japan, Kenya, Norway, and Poland. A total of 834 participants responded and completed the test; 445 respondents (mean age = 27.1; *SD* = 9.5) selected ‘female’ for their gender, and 384 (mean age = 29.0; *SD* = 9.1) selected ‘male’; 5 more selected ‘other’ for gender (mean age = 19.8; *SD* = 1.3). Twelve participants (4 females) were excluded on the basis that they chose incorrectly both the darker face when requested to click on the ‘lighter’ face in a pair and on the lighter face when requested to click on the ‘darker’ face (i.e., in the first and last trials of the test or the “catch” trials; see Fig 2, bottom row). Respondents from Asia Minor, North Africa, and South Asia, were excluded from statistical analyses. Table 1 summarizes results for a total of 24 African females (Mean age = 30.2; *SD* = 9.3), 40 African males (Mean age = 29.4; *SD* = 6.9), 102 Asian females (Mean age = 23.3; *SD* = 10.2), 140 Asian males (Mean age = 26.2; *SD* = 8.7), 248 European females (Mean age = 28.5; *SD* = 8.9), and 168 European males (Mean age = 31.3; *SD* = 9.3). Appendix A includes details of the ethnicity information with our sample.

**Table 1:** Experiment 1. Analyses of choices in the lightness comparison task.

	N	Faces	Question	FRL-bias: Upright faces	p-value (UF)	FRL-bias: Upside-down faces	p-value (UDF)
**African females**	24	African-European	Darker?	20	.001	19	.003
**African females**	24	African-Morph	Darker?	18	.012	14	.270
**African females**	24	European -Morph	Darker?	17	.033	15	.153
**African males**	40	African-European	Darker?	35	.0001	26	.040
**African males**	40	African-Morph	Darker?	30	.001	24	.134
**African males**	40	European -Morph	Darker?	31	.0001	27	.019
**Asian females**	102	African-European	Darker?	72	.0001	65	.003
**Asian females**	102	African-Morph	Darker?	69	.0001	56	.186
**Asian females**	102	European -Morph	Darker?	73	.0001	54	.310
**Asian males**	140	African-European	Darker?	105	.0001	80	.054
**Asian males**	140	African-Morph	Darker?	88	.001	87	.002
**Asian males**	140	European -Morph	Darker?	95	.0001	87	.002
**European females**	248	African-European	Darker?	191	.0001	177	.0001
**European females**	248	African-Morph	Darker?	178	.0001	164	.0001
**European females**	248	European -Morph	Darker?	185	.0001	163	.0001
**European males**	168	African-European	Darker?	134	.0001	120	.0001
**European males**	168	African-Morph	Darker?	130	.0001	117	.0001
**European males**	168	European -Morph	Darker?	124	.0001	101	.005
**African females**	24	African-European	Lighter?	19	.004	17	.033
**African females**	24	African-Morph	Lighter?	19	.004	13	.419
**African females**	24	European -Morph	Lighter?	18	.012	16	.076
**African males**	40	African-European	Lighter?	30	.001	27	.019
**African males**	40	African-Morph	Lighter?	29	.003	25	.077
**African males**	40	European -Morph	Lighter?	32	.0001	28	.008
**Asian females**	102	African-European	Lighter?	82	.0001	61	.029
**Asian females**	102	African-Morph	Lighter?	70	.0001	60	.046
**Asian females**	102	European -Morph	Lighter?	75	.0001	52	.460
**Asian males**	140	African-European	Lighter?	116	.0001	95	.0001
**Asian males**	140	African-Morph	Lighter?	106	.0001	83	.017
**Asian males**	140	European -Morph	Lighter?	101	.0001	81	.037
**European females**	248	African-European	Lighter?	185	.0001	164	.0001
**European females**	248	African-Morph	Lighter?	175	.0001	148	.001
**European females**	248	European -Morph		182	.0001	149	.001
**European males**	168	African-European		126	.0001	101	.005
**European males**	168	African-Morph		116	.0001	103	.002
**European males**	168	European -Morph		121	.0001	109	.0001

The first column shows Ethnic groups, split by Gender. The second column the number (N) of participants in each group. The third column the three types of face pairs (African-European indicates the standard illusion stimuli). Then the type of question posed to the participants (i.e. “which face is darker?’ vs. ‘“which face is lighter?). The last four columns show the counts of occurrences of a Face-Race Lightness Illusion (FRL) Bias response for the Upright Faces (UF) and the Upside-down Faces (UDF) with the corresponding probabilities of outcome (given two forced choices). The p-values indicate the (one-tailed) probabilities in binomial tests, given the sample’s N and the number of choices.

#### Apparatus

We implemented the experiment with *JavaScript* and each participant ran the experiment on their own computer via a web browser, as is typically the case with web crowdsourcing experiments [34].

#### Stimuli and procedure

We used the images shown in Fig 2, which are essentially versions of the original demonstrations [1, 35]. We adjusted the face pairs (except in the “catch trials”) so as to be as similar as possible in their average luminance, contrast (by equating standard deviations in pixels’ brightness), and total numbers of pixels. *Adobe Photoshop CS6* enabled us to make the above adjustments to a high level of precision.

In each trial, the African or European face appeared equally often on the left or right of each other. We also generated upside-down, rotated, versions of the above stimuli without changing any of the image parameters. Additional stimuli included one of the two original ethnicities’ face images together with a 50% morph of the African and European faces, generated with *Morpheus Photo Morpher*, and these also were adjusted to be equiluminant to the other face images. Finally, in the first and last trials of the Internet test, one of the faces in a morph pair was visibly darker or lighter. These constituted “catch” trials aimed to verify that participants followed the specific instructions that appeared on screen.

At the end of the perceptual trials, participants continued by filling out a questionnaire, where they could, either by selecting within a pop-down window or clicking on boxes, select specific characteristics (e.g., gender) and, for the ‘open’ questions, type in answers. The goal of the questionnaire was to collect basic demographic information about each participant, while maintaining anonymity, as well as to probe whether the participant guessed the purpose of the experiment. The items were in order of appearance: a) Select your country (a pop-down window allowed to select from a list of all world countries); Select your Ethnicity (by clicking within boxes labeled: African, Asian, European, Other; with option of typing in a name when this is selected); b) Select your Gender (Female, Male, Other); Your Age (type a number); Years spent in current country (boxes: All my life; less than a year; 1–5 years: 6–10 years; 11–15 years; 16 years or more); ‘what do you think was the purpose of this test?’ (Open answer).

### Results

First, we computed the prevalence of the FRL bias from responses in the two ‘target’ trials, these corresponding to the standard demonstrations of the face-race lightness illusion (i.e., the African and European faces in Fig 2, top row), based on whether respondents selected a) the African face when queried about “which face looks darker?” and b) the European face when queried about “which face looks lighter?”. In order to examine responses from the whole sample, we initially ignored the demographic characteristics of the participants and found that 78.3% (*SD* = 29.2) of total responses were consistent with the FRL illusory bias. Specifically, out of 822 respondents, a total of 504 (61.3%) made selections consistent with the FRL bias in both target trials, whereas 279 participants (33.9%) answered according to the bias in one trial but in not the other. Finally, 39 participants (4.7%) responded in the opposite direction to the bias in both trials (i.e., click on the European face for “which face looks darker?” and the African for “which face looks lighter?”). In trials where one of the target faces was paired with a 50% morph of ambiguous ethnicity, the FRL bias was still present in both cases (African face + Morph: 66.8%, *SD* = 44.7; European face + Morph: 68.3%, *SD* = 44.7), although slightly reduced compared to the standard face pair (72.4%, *SD* = 46.5). In general, inverting the orientation of the face reduced the FRL bias, since this occurred in 74.8% of all trials, whereas when the same stimuli were seen upside-down the bias was present in 63.6% of trials. The prevalence of the bias across participants’ gender appeared to be similar across all trials (women: 69.24% of trials, *SD* = 46.2; men: 69.22% of trials, *SD* = 46.2).

A preliminary analyses revealed a highly similar occurrence of the bias the ‘darker?’ and ‘lighter?’ questions, (‘darker?’: 70%, *SD* = 45.8; ‘lighter?’: 68%, *SD* = 46.5) and, consequently, the following analyses are based on collapsing choices in both conditions. A 6x2 Chi-square analysis on the number of FRL ‘bias’ responses for the standard illusion stimuli (i.e., the African and European upright face pairs), with Groups (African females, African males, Asian females, Asian males, European females, European males) as independent variables, showed no significant difference in frequency of FRL bias, Chi-square: 1.95, *df* = 5, p = 0.85. An analogous 6x2 Chi-square analysis on the number of FRL ‘bias’ responses for the upside/down version of the African and European face pairs, with Groups (African females, African males, Asian females, Asian males, European females, European males) as independent variables, also failed to show any significant difference in frequency of FRL bias, Chi-square: 3.17, *df* = 5, p = 0.67.

Finally, we examined the open answers to the last item in the questionnaire where participants had typed in freely their answers to the question “what do you think was the purpose of this test?” In order to identify participants who were either already aware of the existence of the face-race lightness illusion or guessed it correctly, we searched through the whole sample for responses mentioning at least once the following ‘key’ words: illusion, bias, prejudice, skin, color (or colour for British English spelling). Among the Africans, 1 participant mentioned ‘illusion’, 11 mentioned ‘color’, 12 participants mentioned ‘skin’, none wrote ‘prejudice’, and 1 used the word ‘bias’. Among the Asians, 8 participants mentioned ‘illusion’, 25 mentioned ‘color’, 5 mentioned ‘skin’, 1 mentioned ‘prejudice’, and 1 used the word ‘bias’. Among the Europeans only 2 participants mentioned ‘illusion’, 98 mentioned ‘color’, 121 participants mentioned ‘skin’, 6 mentioned ‘prejudice’, and 9 used the word ‘bias’. Note that, across the whole group, skin and color were combined together in 156 responses and that 17 participants used the word ‘same’ in combination with either skin or color, indicating that they noticed that most face pairs had the same luminance (e.g., “Every people on pics have the same color of skin”; as in an anonymous answer to the open-ended question). Nevertheless, it is apparent from all open answers that many participants did not seem to have an idea of what the test was about since they simply left as response the default option: “none” (N = 224), and some explicitly wrote “don’t know” (N = 20) or “no idea” (N = 20). In sum, few out of the 822 participants seemed to be explicitly aware of the existence of an illusion or a bias linked to ethnicity and only a few reported that the skin tones looked the same. Although a minority mentioned either color or skin (often together), this does not incontrovertibly indicates that they understood the purpose of the test, since the task demands were to explicitly choose the faces with darker/lighter skin tones.

### Discussion

The main conclusion from the present web-based experiment is that the race-face lightness illusion is replicable across different geographical locations and participants’ ethnicities. However, it also emerges that not every participant shows the FRL bias and about a quarter of the respondents may not experience a visible difference between the two faces, thus distributing their responses more evenly between the African and European faces in the present forced-choice test. It is also important to point out that more than half of the respondents in the present sample were from European countries, hence from so-called WEIRD societies [14], however about 10% of the respondents shown in Table 1 were from less affluent countries in Africa (e.g., Nigeria) and about one third of all participants were from non-Western, but affluent, East Asian countries (e.g., Japan).

It was also clear that posing the question as a ‘darker’ or ‘lighter’ luminance judgment did not affect the probability of the illusory perception. As indicate in Table 1, the African and European face pair yielded the expected bias within all groups, irrespective of ethnicity or gender and to some extent also when seen reversed. However, showing the face stimuli upside-down did reduce the presence of the FRL, especially for the African and Asian groups. Indeed, all of the observed non-significant results occurred in the reversed faces conditions.

Remarkably, the present results do not particularly support the ‘artifact-based’ account, which posits that the FRL effect is neither dependent on identifying the ethnicity of the stimuli or on the ethnicity of the observers, but is rather dependent on low-level properties of the stimuli per se. In fact, we observed a reduction of the bias when the face stimuli were presented upside-down, consistent with the idea that this manipulation impedes the ability to recognize the faces’ ethnic identities [3, 7, 31]. Moreover, pairing each face with an ethnically ambiguous face (i.e., the 50% morph of the African and European faces) appeared to lower the occurrence of the bias.

## Experiment 2

**Fig 3:**
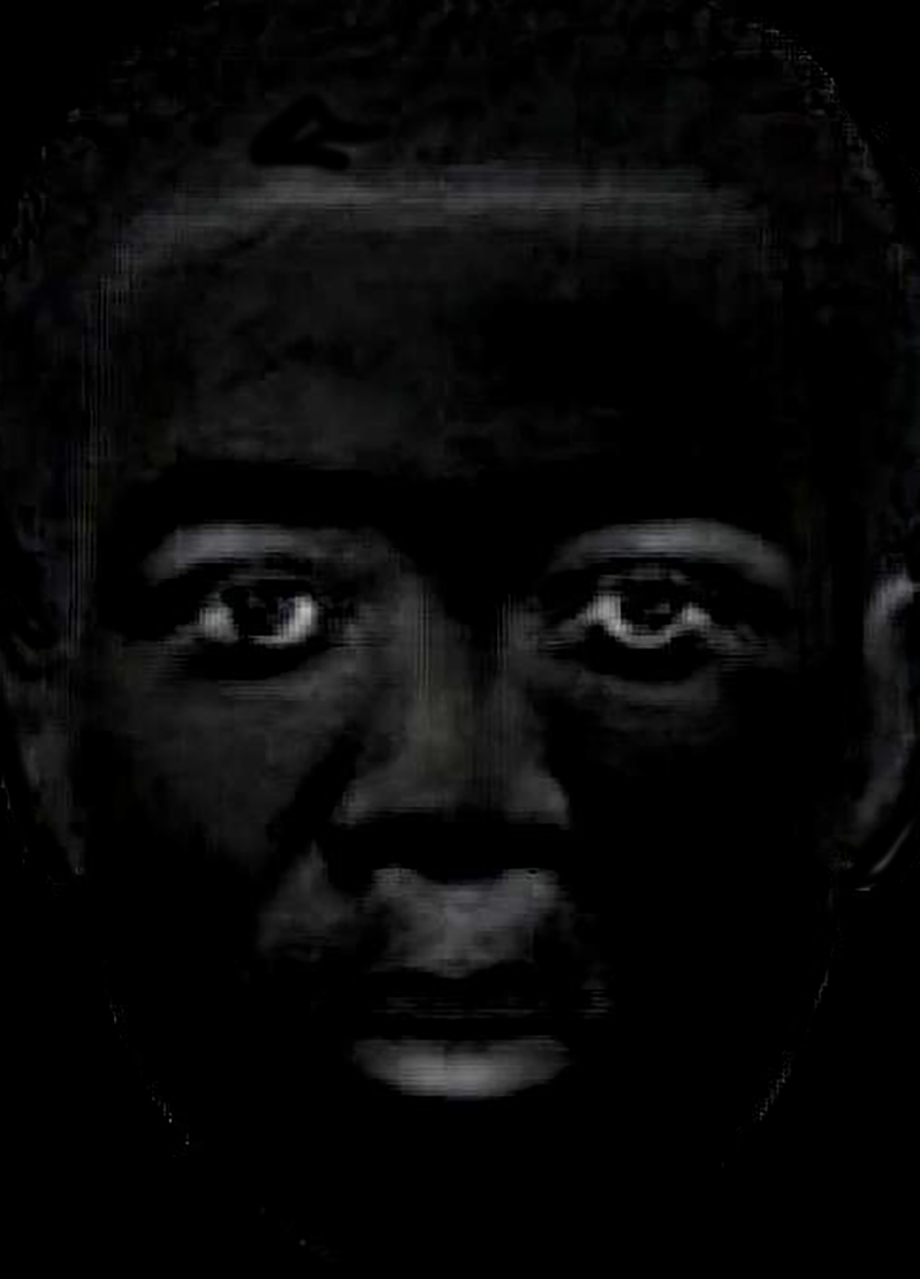
Local regions of brightness difference between the African and European face stimuli used in Experiment 2. Brighter regions indicate more luminance in the African’s than the European’s facial image.

Although the faces used in the FRL illusion are, by definition, equal in mean luminance, in all known demonstrations, gaze is free to move and scan the images, so that different parts or local regions of the faces might be attended overtly. In fact, equal average luminance does not mean that local intensities do not differ in the two images, since this happens only when the two images are identical. As noticed by Firestone and Scholl [27], the nose of the African face used in several demonstrations of the illusion appears of a lighter tone than the European’s nose. In order to make explicit regions of maximal differences in brightness between the two faces, we subtracted the pixels’ intensities of the latter from the former using the MATLAB *imabSDiff* command (see Fig 3). This procedure revealed that the African face looked, at visual inspection, indeed brighter than the European face in several regions, namely: the forehead region bordering the hairline, the eyebrows, the sclera of both eyes, the bridge of the nose and the region immediately below the nostrils and above the mouth, as well as the lower mouth lip.

Hence, it is entirely possible that in standard demonstrations of the illusion, observers may tend to fixate on parts of the African face that are saliently dark, as pointed out by Firestone and Scholl ([28], p. 13), while doing the converse with the European face and fixating on parts of the face that are saliently bright. That is, differences in attentional control or overt shifts of the eyes could change what we see by sampling different input.

In general, research employing eye-tracking has revealed that there are differences in the way observers from different culture look at own- and other-race faces [36–38], although some studies indicate that such differences can be rather subtle [39]. We surmise that even small changes in dwell time of gaze on darker vs. brighter regions of a stimulus might be sufficient to evoke noticeable differences in perceived luminance. Hence, in the second experiment, we directly registered oculomotor behavior on the two faces of the illusion.

In Experiment 2, we also made an effort to include participants of different ethnicities in the laboratory, by recruiting not only Norwegian participants at the University of Oslo, but also a group of African students from Kenya, who were all born and raised in Africa and had been residents in Norway only for their period of study at University of Oslo. In addition, we tested a group of East Asian university students at Senshu University in Tokyo (Japan). Importantly, the cognitive laboratory at Senshu is equipped with the same eye-tracking system (SMI) at the laboratory of University of Oslo; hence the experimental conditions, procedures and quality of data collection was extremely similar across laboratories and the only planned difference was the ethnicity of the participants.

**Fig 4:**
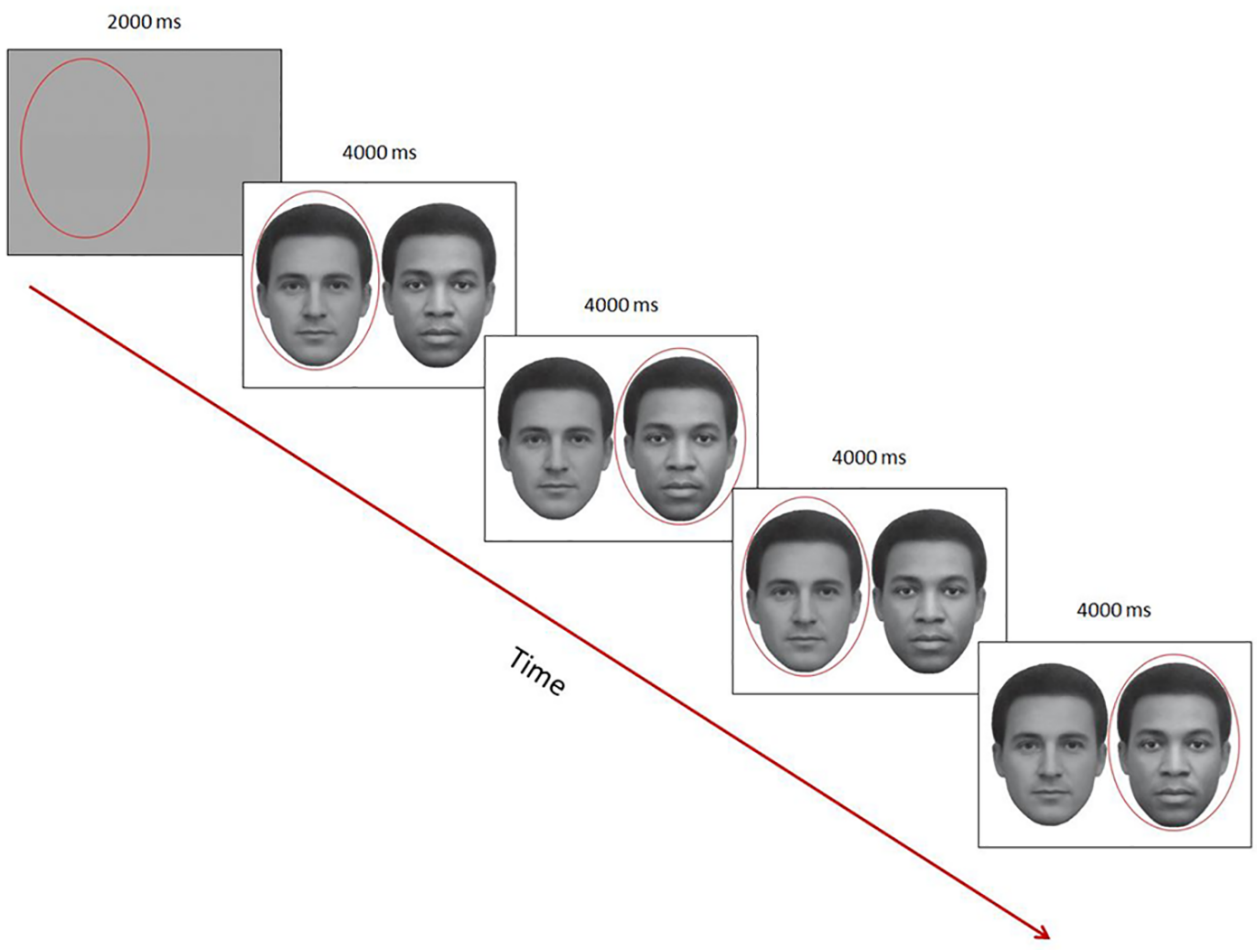
Timeline of one trial in Experiment 2: Participants maintained gaze within the red ellipse at all times. The red ellipse worked as a cue for keeping gaze (every 4 secs) within one of the regions containing only one of the two faces. A trial began with a neutral blank gray image, equiluminant to the average brightness of the successive faces image, and serving as a baseline image to compute pupil changes in an event-related manner (by subtracting the mean pupil diameter during viewing of each baseline image from the mean pupil diameter when subsequently attending a face stimulus).

Specifically, participants viewed repeatedly, on a computer screen, side-by-side face images of an African male and European male, in an analogous manner as when testing for the presence of the FRL bias in Experiment 1. However, a major difference in this experiment was that, before presenting a face pair, a colored ellipse would ‘cue’ for one second the participants’ gaze within one region of the blank screen (see Fig 4 for illustration) that corresponded to the location of one of the two faces during the stimulus presentations. Subsequent to the cue, we presented a face pair and each participant maintained gaze, at all times, within the ellipse, thus constraining their ‘overt attention’ to only one face of the two simultaneously presented faces. Although the locus of fixation is not always synonymous with the direction of covert attention [40, 41], covert shifts of attention are closely related to internal switches of attention and they typically operate as preparatory mechanisms for the control of overt shifts of gaze [42, 43]. If shifts of attention normally precede shifts of the eyes to the same location, we would think that spontaneous eye movements provide a very close estimate of what is attended during a period of time [44] or, as put by Findlay ([45], p. 136), that “eye scanning typifies the way visual attention is normally deployed.”

Note, however, that we did not force gaze on any specific part of the face and participants could freely scan the faces included within the ellipse. After 4 seconds, the ellipse shifted to the other side enveloping the other face, allowing attention and focal vision to examine with gaze the face that previously was visible only peripherally. We repeated such an alternation of side of focus twice within each trial so that we monitored 4 times in a row a participant’s gaze (and pupil) while it remained onto each face in a pair.

By “forcing” gaze (but not fixation) on one face and then the other, for identical amounts of time, we ensured that the measured pupillary changes within each 4 sec epoch were related to looking at one specific stimulus (face) at a time. In general, when applying pupillometry to images with several simultaneously presented objects (even just two objects as in the present study), it is difficult to attribute to a particular item the measured pupillary change, since gaze can move rapidly from one object to another. Hence, it may be difficult to tease apart the pupillary effect of the presently fixated stimulus and locus of gaze from that of the immediately preceding item, especially when observers can move their eyes freely over several items within the whole scene. Moreover, it is known that pupil diameter can increase or decrease with luminance of what is fixated as well as to a lower extent of the surround. These luminance-related responses are called the ‘pupillary light reflex’ and ‘darkness reflex’ and both have similar onset latencies of about 200 milliseconds, though the darkness response’s peak tends to be slower and weaker in amplitude than that caused by light increases [46, 47]. In contrast, pupillary changes (typically dilations) to cognitive factors can have a longer time lag of about half a second to 1 second [48–51].

According to the ‘Artifact’ account, one would expect that eye fixations would differ when attending to the African versus the European face and that this would be driven by salient, low-level or sensory features of the stimuli. Specifically, Firestone and Scholl [27, 28] suggested that attention may be captured within the African face by a salient region of contrast and darkness in the lower part of the face (corresponding to the mouth and jaw), thus increasing the likelihood of seeing this face as darker than when looking at the corresponding region in the European face. Based on the above pupillary dilations to darker and constriction to lighter stimuli, one would also expect that the hypothesized (artifactual) gaze biases would necessarily result in larger pupils when attending the African face than when attending the European face. The stereotype-based account would make a similar prediction, but for different reasons; that is, an enlarged pupil when attending the African face could reflect emotional reactions [52], especially in individuals with high scores in prejudice measurements. Hence, we tested for the present experiment a subgroup of participants for prejudice towards skin tone by use of the Implicit Attitude Test or IAT [53]. Although the original study by Levin & Banaji [1] had failed to reveal a relationship between strength of the FRL and measures of ‘explicit’ prejudice, they did not exclude the possibility that attitudes that are either unknown to the participants or difficult to censor consciously can play a role in the responses to the illusion. Indeed, an fMRI study [54] found greater amygdala activation for African than European faces when faces were presented subliminally (for only 30 ms and masked), and activation was stronger the higher participants’ implicit racial bias (on the IAT).

The ‘Prototype-based’ account [1] did not originally generate any predictions about either eye fixations or pupillary size; although we surmise that this account can lead to expect pupillary changes consistent with the distorted percept. That is, if looking at the African face results in a darker percept than for the European face, then one could predict the pupils should adjust over time, due to the influence of the top-down distortion of representations of the faces in visual cortex. It is known that internally generated quasi-perceptions or “mental images” have similar neural representations to a corresponding object when perceived by the senses [55]. Imagined objects or events can trigger pupil responses in a similar manner to actual input or perceptual stimulation with the same objects and, most relevantly, imagining objects that are bright or dark induces the diameter of the eye pupil to match the content of the mental scenario [56, 57].

Finally, the ‘Predictive sampling’ account would seem able to generate different predictions about eye fixations and pupil size than other accounts. Specifically, as for the Artifact account, eye fixations should differ for the two faces, but not necessarily by focusing on darker face regions for the African face, or lighter regions for the European face. In fact, it can predict the opposite behavior: if gaze fixated the lighter regions of the African face and/or the darker regions of the European face, this could cause the attended African face to look darker while the European face would look lighter. That is, when pupils are relatively smaller, this reduces retinal illuminance, while when they are relatively larger retinal illuminance increases [58]. The net effect would be a change in the intensity of light energy entering the eye in each of the two cases, associating differentially bright/dark percepts with each face. This specific prediction makes the joint measurement of fixations of gaze and pupils diameters particularly valuable, if not crucial, in order to identify key mechanisms underlying the FRL bias.

Given that people are often prejudiced against “out-group” individuals [53], it is also possible that pupil responses reflect the spontaneous emotional reactions to simply viewing out-group faces. Interestingly, when pictures of biracial politicians (e.g., Barack Obama) were shown with altered skin tones (either darker or lighter), participants judged the “lighter” versions as more representative than darker versions of the same face [24]; especially when the observers were partisans to the portrayed politician. Thus, it would seem that stereotypes could affect the perception of skin tones, a conclusion that would be consistent with what we labeled the Stereotype-based account. However, in the original study by Levin and Banaji [1], scores in a questionnaire about explicit attitude to skin color failed to reveal any significant (positive) relationship to the strength of the illusion. Because explicit attitudes could be self-censored, measures of “implicit attitudes” [53] may be more likely to relate to pupil responses. Note also that pupil responses occur automatically and they can be considered an “honest signal” that observers cannot suppress or easily generate at will [56]. Hence, in the present experiment, several participants also completed the online Implicit Association Test (IAT) after the eye-tracking session.

### Methods

#### Participants

These were university-level students enrolled at Universities and Colleges in Norway (principally University of Oslo) or at Senshu University, Tokyo, Japan, who all volunteered for a study advertised as study on eye movements on faces. They did not receive payment for their participation. Participants belonged to three ethnic groups: Africans (N = 22; 11 females, mean age = 27.1, *SD* = 5.2); Asians (N = 24; 12 females; mean age = 26.3, *SD* = 6.4); and Europeans (N = 35; 20 females; mean age = 25.4; *SD* = 5.2). They all had normal or corrected-to-normal visual acuity by use of contact lenses. All the Africans students were originally from Kenya and had lived in Norway as students for a short period (range: few weeks to 2 years). All participants agreed to an informed consent and were treated and in accordance with the Declaration of Helsinki. The study was ethically approved independently by the Institutional Care and Use Committee of the two Departments of Psychology in Japan and Norway; that is, the Institutional Ethics Committee at Senshu University (Japan) and the Internal Research Ethics Committee PSI at the University of Oslo (Norway).

#### Apparatus

For both the Oslo and Tokyo laboratories, the displays were set at 1280 x 1024 pixels and presented on 47 cm, flat, LCD monitors. Both experimental sessions in Norway and Japan took place in rooms with constant illumination, kept at standard levels throughout testing sessions. Crucially, we collected in both labs the oculomotor data by using two machines of same model: the Remote Eye tracking Device 250 (RED), both built in the same year by *SensoMotoric Instruments* (SMI; Berlin, Germany). RED has an automatic compensation for head movements within 0.7 m distance and within a range of 40x20 cm. The sampling rate was set in both cases at 60 Hz and *BeGaze* software (SMI) was used afterwards to automatically detect fixations whenever gaze dwelled for a minimum of 80 ms within a region of maximum 100 pixels, following a standard algorithm.

#### Stimuli and procedure

The stimuli consisted of the standard face pairs of an African and European male (as in Fig 1), that is images of non-existent individuals or morphs where the ethnicity of the individuals is clearly visible and that have resulted in the classic FRL illusion in previous studies (e.g., [1]). However, we edited the original face images in *Adobe Photoshop* and re-sized them so that each face image had a similar amount of pixels’ count and were as close as possible in luminance (by adjusting their pixels’ brightness in HSL/RGB units: Mean Luminance = 112.5 ± 1). In the experiment, the standard pair of faces was presented four times (half of the time with the African face on the left side). The same stimuli were shown twice upside-down, counterbalancing left/side positions of the face ethnicity. There were also six filler stimuli, where either one or both faces were shown as uniform gray within the face outer contour, i.e. with no internal facial features visible. All stimuli were presented in a fixed, pseudorandom, sequence.

At the very beginning of each session, both in Oslo and Tokyo, participants followed a 4-point calibration routine. Following this, a face pair was displayed for a total of 16 seconds, while a shifting cue informed participants, every 4 seconds and twice in a trial, to keep gaze within the elliptical cue surrounding a face (as illustrated in Fig 4). The African face appeared on the left side or on the right side in an equal number of trials. The position of the initial cue varied unpredictably across trials but attention was first cued equally often to the African or the European face. In two additional trials, participants saw the same face pairs rotated upside down, also alternatively cued by a surrounding ellipse. No explicit response (verbal or key press) was required during the viewing of the stimuli. Between these ‘target trials’ (upright, reversed) there were pairs of filler face stimuli, also luminance-matched with the target images to avoid step changes in luminance and in turn sudden changes in pupil diameter along the experiment. At the end of the experiment, we collected standard demographic data (e.g., age, sex). About half of the participants in each ethnicity group had also time to complete the on-line *Implicit Association Test (IAT*: https://implicit.harvard.edu/implicit/takeatest.htm). The IAT task entails grouping of words and images into categories. At debriefing, none of the participants reported having previous knowledge about the face-race lightness illusion or that they perceived the two noticed that they grey-tone faces had the same luminance.

### Results

#### Eye fixations

**Fig 5:**
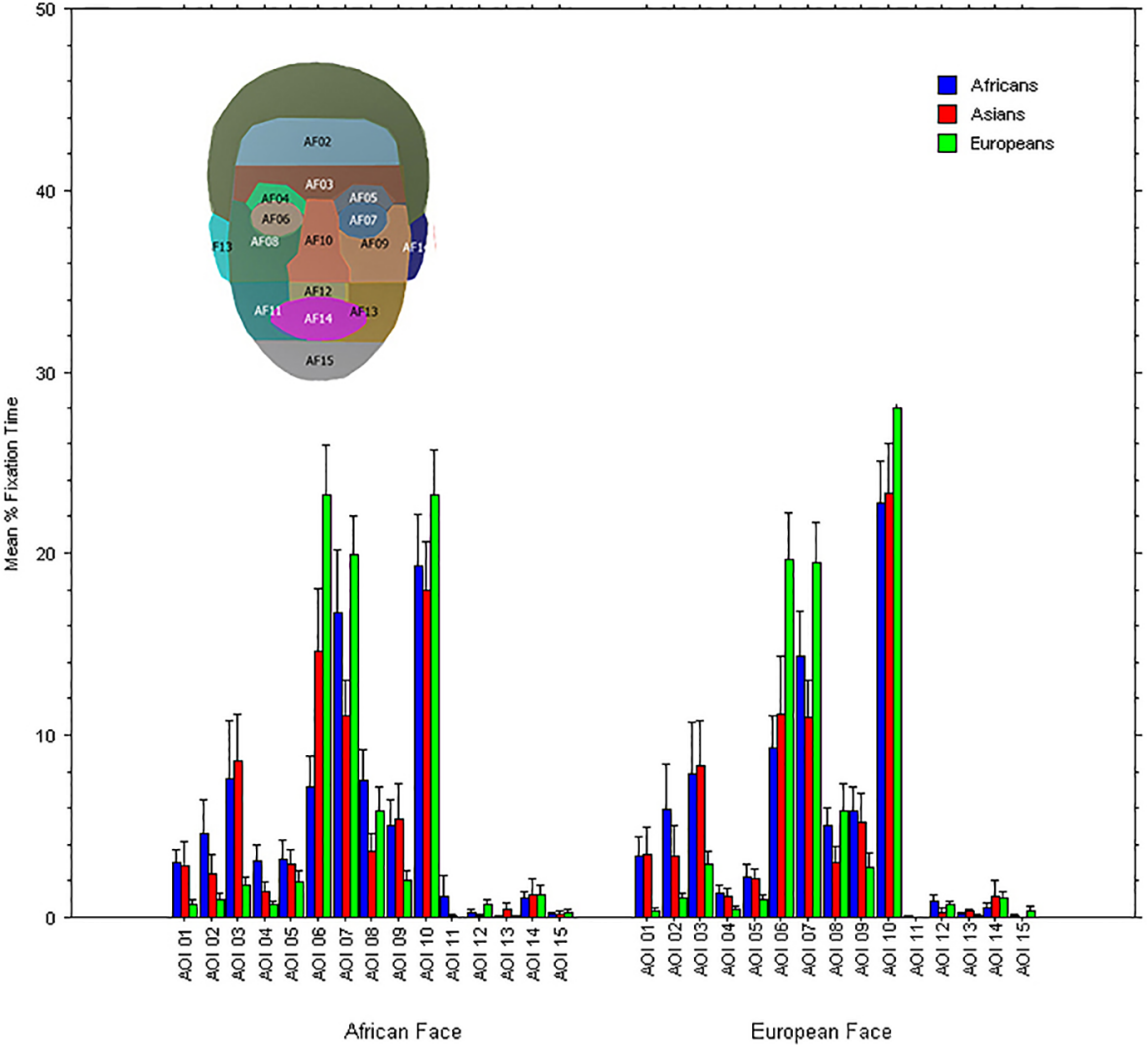
Mean % fixation durations (Y axis) within different Faces regions (AOI) for the three groups of participants when viewing each upright face: Africans (blue columns), Asians (red columns), and Europeans (green columns). Error bars represent Standard Errors. In the inlay the face regions corresponding to each AOI (the colors are arbitrary) are shown superimposed to the “African” face.

To assess differences in dwell times of gaze, we first created 15 Areas of Interest (AOI) corresponding to salient and nameable regions of the face (see face inlay of Fig 5), several of which also differed in shading (as revealed in Fig 3). The top-positioned AOI-1 included hair, whereas the lowest-positioned AOI-15 included the chin. Also, based on the previous literature on attention to faces of different races [59], one could expect that the nose area might be particularly relevant when comparing African and European faces; hence, one AOI (10) outlined the nose in each face. We then computed the mean percentage fixation time spent within each AOI, for each participants and each face ethnicity, by use of *BeGaze* software (by SMI). We note that when computing the average pupil diameter during fixations only, such an algorithm automatically removes artifacts as eye blinks and avoids including pupil measurements while the eye is travelling (i.e., during saccades).

We first reasoned that a random distribution of gaze over the face would result in a dwell time of 6.66% of time (i.e., 100/15) within each AOI. Hence, dwell times that do not differ from this base rate, for at least one of the two faces, are not considered meaningful for assessing attentional differences. We therefore computed 95% confidence intervals of mean percent dwell times for each AOI and for each face and each group of participants. As it can be seen in Fig 5, many AOIs were not looked at significantly above chance, including several corresponding to putative differences in luminance between these face images (e.g., mouth, jaw), also consistently across the three Ethnicity groups. Instead, it was clear that the AOI-6 and AOI-7 (corresponding to the left and right eye) and AOI-10 (corresponding to the nose) were looked for lengths of time that were well above chance and, again, by all groups.

Therefore, after summing the percent dwell times within the AOIs of the eyes, we performed a repeated-measures ANOVA with Participants’ Ethnicity (Africans, Asians, Europeans) as between-subject factors and Face Ethnicity (African face, European face), Face Parts (Eyes, Nose) and Orientation (Upright, Upside-down) as within-subject factors. This revealed a main effect of Participants’ Ethnicity, *F* (2, 78) = 18.99, *p* < 0.0001, η^2^_p_ = 0.3. This effect was accounted by longer dwell times of the European participants on the eyes and nose (mean dwell time = 33.0%) than for either African or Asian participants (mean dwell time = 22.5%, and mean dwell time = 22.4%, respectively). There was also a main effect of Face Ethnicity, *F* (1, 78) = 7.07, *p* = 0.01, η^2^_p_ = 0.1. The European face was looked for a greater percent of time (mean dwell time = 27.8%) than the African (mean dwell time = 26.1%) on these face parts. The other main effects did not reach significance (0.29 < p < 0.87). Importantly, the factor of Orientation interacted significantly with Face Parts, *F* (1, 78) = 5.14, *p* = 0.026, η^2^_p_ = 0.1, and with both with Face Ethnicity and Parts in a three-way interaction, *F* (1, 78) = 8.21, *p* = 0.005, η^2^_p_ = 0.1.

Given that Orientation interacted with the ethnicity of the faces and their parts, to better understand the patterns of results, we further analyzed these results in two separate repeated-measures ANOVAs, with Participants’ Ethnicity (Africans, Asians, Europeans) as between-subject factors and Face Ethnicity (African face, European face), Face Parts (Eyes, Nose) as within-subject factors.

**Fig 6:**
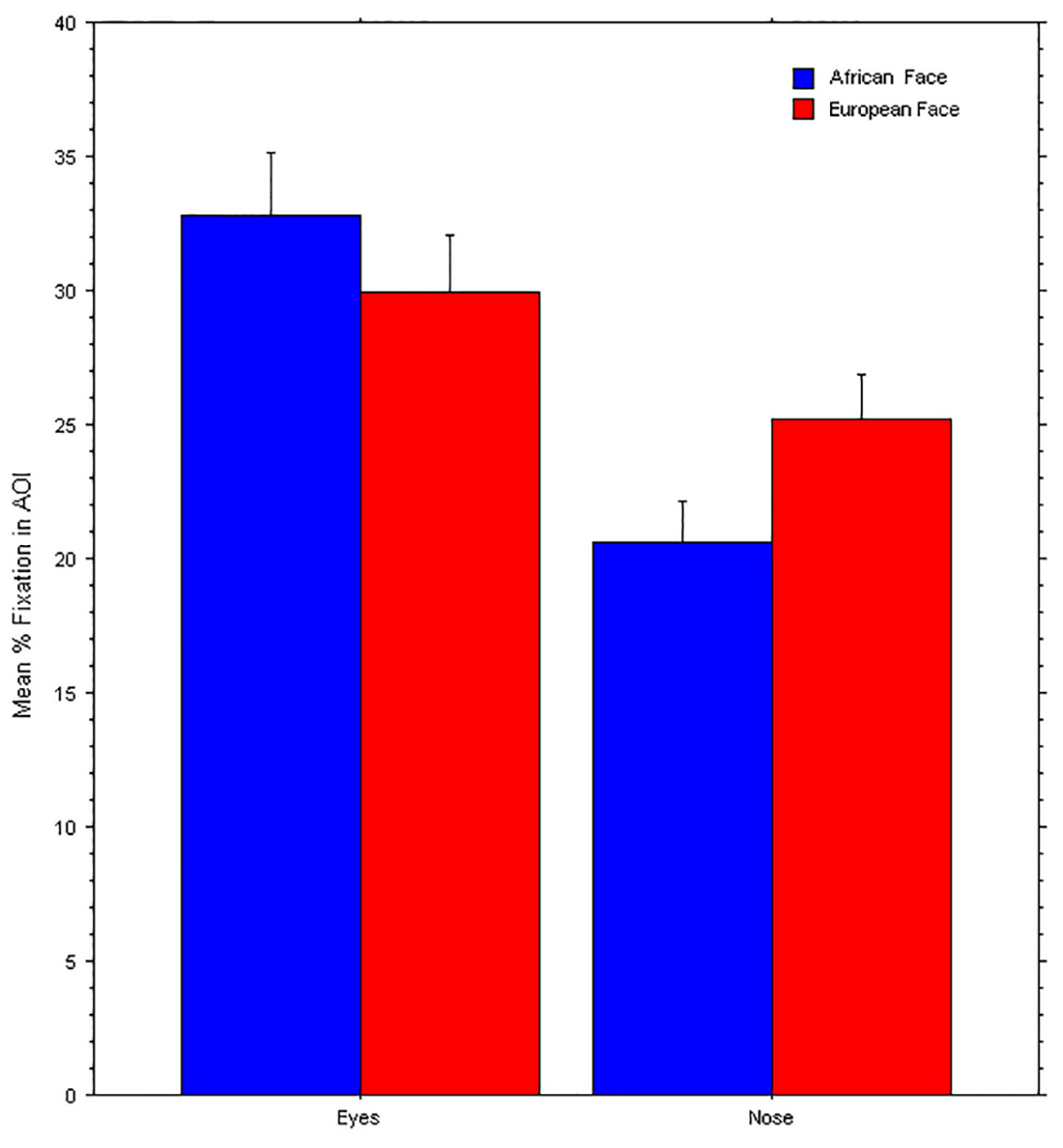
Mean % fixation durations (Y axis) or dwell time within different faces parts (Eyes, Nose) when viewing the upright African face (blue) and European face (red). Error bars represent Standard Errors.

The repeated-measures ANOVA on ‘upright faces’ revealed a main effect of Participants’ Ethnicity, *F* (2, 78) = 16.73, *p* < 0.0001, η^2^_p_ = 0.3. This effect was accounted for by longer dwell times of the European participants on the eyes and nose (mean dwell time = 33.0%) than for either African or Asian participants (mean dwell time = 22.5%, and mean dwell time = 22.4%, respectively). There was also a main effect of Face Parts, *F* (1, 78) = 5.46, *p* = 0.022, η^2^_p_ = 0.1. The eyes were looked at for a greater percent of time (mean dwell time = 31.5%) than the nose (mean dwell time = 23.0%). Importantly, there was a significant interaction between Face Ethnicity and Face parts, *F* (1, 78) = 16.73, *p* < 0.000, η^2^_p_ = 0.2. As shown in Fig 6, while the eyes were attended to relatively more in the African face than in the European, the nose was attended to relatively more in the European face than the African. No other effects reached significance (0.08 < p < 0.65).

The repeated-measures ANOVA on ‘upside-down faces’ revealed a main effect of Participants’ Ethnicity, *F* (2, 78) = 9.68, *p* = 0.002, η^2^_p_ = 0.2. This effect was again accounted for by longer dwell times of the European participants on the eyes and nose (mean dwell time = 31.9%) than for either African or Asian participants (mean dwell time = 23.4%, and mean dwell time = 22.5%, respectively). There was also a marginally significant main effect of Face Ethnicity, *F* (1, 78) = 4.29, *p* = 0.042 η^2^_p_ = 0.01; the European Face’s Eyes and Nose were examined for a greater percent of time (mean dwell time = 28.0%) than those of the African Face (mean dwell time = 25.5%). We again found a significant interaction between Face Ethnicity and Face parts, *F* (1, 78) = 24.02, *p* < 0.0001, η^2^_p_ = 0.1. This interactive effect was due to participants centering their gaze the most on the nose of the European Face (mean dwell time = 33.2%) and the least on the African nose (mean dwell time = 21.1%); again, eyes were attended to relatively more in the African face (mean dwell time = 29.9%) than in the European face (mean dwell time = 23.1%). No other effects reached significance (0.58 < p < 0.78).

#### Pupillary changes

We obtained by use of *BeGaze* (SMI) all mean pupil diameters (in mm) during each eye fixation from each individual and these were averaged for each individual and for each condition (e.g., attending the African face). We first subtracted pupil size during the initial cue presentation, using it as a baseline image to compute pupil changes in an event-related manner. We performed an initial repeated-measures ANOVA with Participants’ Ethnicity (Africans, Asians, Europeans) as between-subject factors and Face Ethnicity (African face, European face) and Orientation (Upright, Upside-down) as within-subject factor.

This revealed a main effect of Orientation, *F* (2, 78) = 19.24, *p* < 0.0001, η^2^_p_ = 0.2, as well as an interactive effect of Orientation with the Face Ethnicity, *F* (2, 78) = 5.77, *p* = 0.02, η^2^_p_ = 0.1. No other effects reached significance (0.12 < p < 0.82). Post-hoc comparisons revealed that the pupils were the least dilated to the European face seen upside-down (p_bonf_ = 0.001). Again, given that Orientation had a major influence on the pupil and modulated the effect of ethnicity of the face stimuli, we analyzed in two separate repeated-measures ANOVAs the pupil diameters when viewing the upright (standard) face images and when the same stimuli were seen upside-down.

The separate ANOVA on mean pupil changes for ‘upright faces’ revealed a significant main effect of Face Ethnicity, *F* (1, 78) = 4.16, *p* = 0.045, η^2^_p_ = 0.1, indicating that the pupil diameters were significantly larger for the European (mean pupil change = 0.13 mm; *SD* = 0.17) than for the African face (mean pupil change = 0.12 mm; *SD* = 0.16). Thus, pupils dilated more (Cohen’s *d*: 0.22) for the “subjectively brighter” (European) face than for the “subjectively darker” (African) face in the FRL illusion. The difference in pupil size corresponded to an 8% increase in pupillary dilation. There was also a significant interaction between Participants’ Ethnicity and Face Ethnicity *F* (2, 78) = 3.24, *p* = 0.044, η^2^_p_ = 0.1, and post-hoc analyses revealed that pupils were significantly more dilated to the African face than the European face for this group of participants than for the other groups.

The original accounts of the illusion assume that the effect depends on identifying first the ethnicity of each of the two faces, followed by a perceptual distortion process and/or differential visual sampling of each stimulus. All of these processes would allegedly take time, though it is unclear what their time-course would be. Hence, it is crucial to compare changes of the pupil diameters over time. Specifically, in order to assess when pupillary diameters differed for the two faces while viewing the illusion, we computed each ethnicity group’s 95% confidence intervals for the moment-to-moment evolution of the pupil changes according to the formula for within-subject design by Loftus and Masson [60, 61].

**Fig 7:**
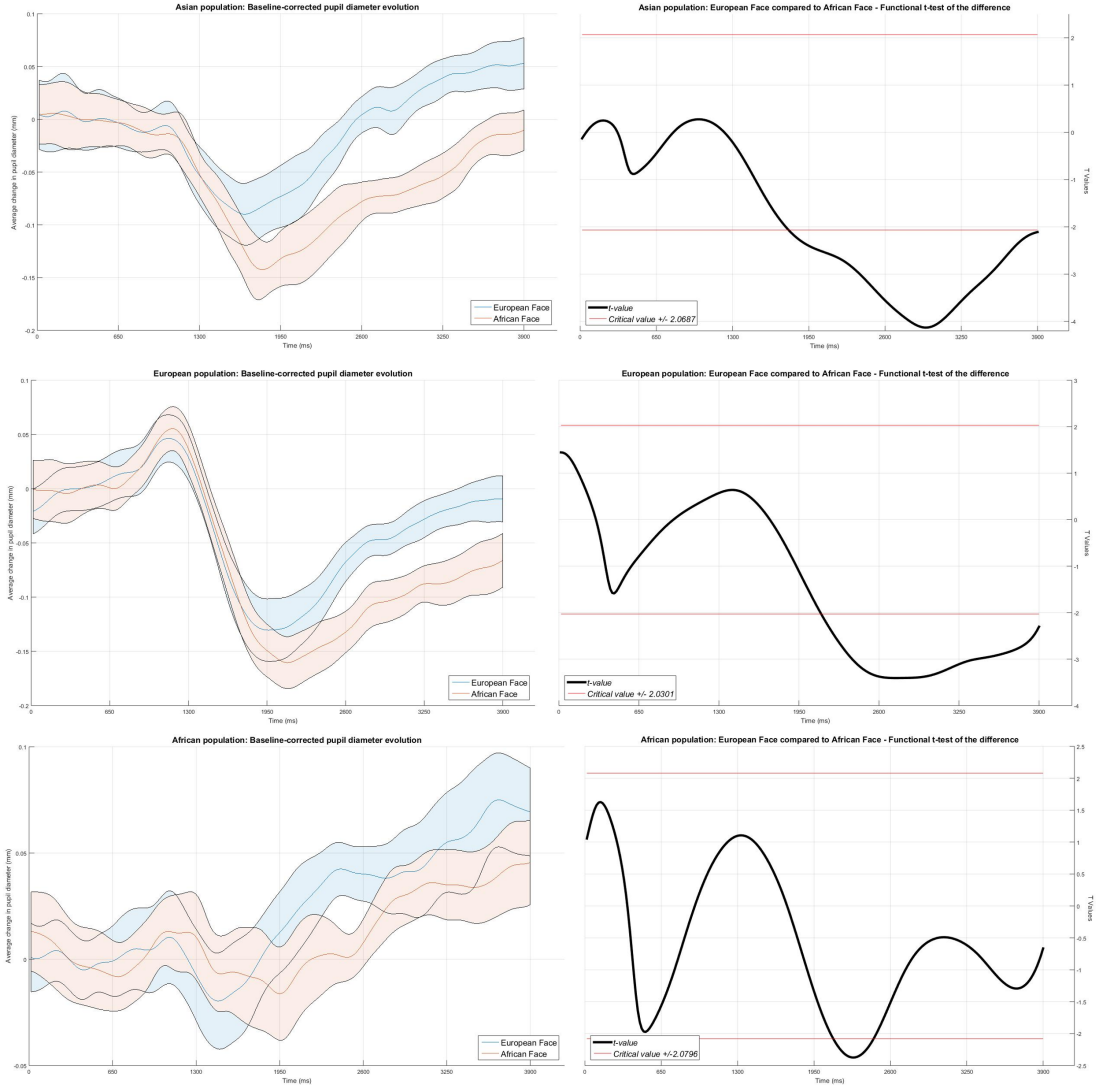
Left panels: Mean baseline-corrected pupillary diameters of Asian participants (Top panel; N = 24), European participants (Middle panel; N = 38) and African participants (Bottom panel; N = 22), evolving over a time period of 4 seconds from onset of the cue around a face, i.e. while attending the “African” face (red line) or the “European” face (blue line). The colored stripes indicate 95% confidence intervals for within-subject comparisons. Right panels: Functional t -tests of the difference between pupils when attending to the African versus European face. The solid (red) horizontal lines represent the two-tailed critical value for t.

We show the resulting curves and intervals in Fig 7 in the left panels. In addition, in the right panels, we show graphs illustrating temporal *t*-test analyses, based on ‘functional data analysis’ (FDA; [62]), where the pupillometry data are transformed into curve functions and statistical analyses are performed on those very functions (for applications of FDA specific to pupillometry, see [63]).

By illustrating the pupillary evolution and associated FDA *t*-test curve for the Asian participants (Fig 7, top panel), it appears that pupil responses to the faces appear very similar until around 1.5 sec, but this group’s pupils also began to dilate more when attending to the European face and, at about 1.7 sec from onset, the pupillary response to the European face became significantly larger than that to the African face and remained so for the rest of the recording epoch.

The pupil diameter evolution graphs and associated FDA *t*-test curve for the European participants only show (Fig 7, middle panel) that pupil responses to the faces were also very similar after onset until around 1.5 sec, this group’s pupils also began to dilate when attending to the European face and, at about 1.8 sec from onset, the dilation to the European face became significantly larger than that to the African face and remained so for the rest of the recording epoch.

The pupillary diameter evolution graph and associated FDA *t*-test curve for the African participants (Fig 7, bottom panel) also shows that pupil responses to the faces were similar after onset until around 2 sec, where the difference between changes of the pupils reached significance for a short period of about 300 ms. Note however, that the pupillary change curve (left panel) shows a similar profile for the rest of trial as for the other groups in Fig 7, with greater dilation of the pupils when attending to the European face compared that to the African face.

Finally, given that pupil differences increased significantly for all groups within 2000 ms and 4000 ms (Fig 7), we checked whether dwell time on the eyes and nose of the two faces differed within this time window compared to the first half of the presentation (i.e., from onset until 2000 ms). For this purpose we first calculated a dwell-time difference score, by subtracting the percent of time spent looking within the previously defined area of interest corresponding to the nose from the eyes’ AOIs. In this manner a positive score would indicate more time spent, relatively, looking at the eyes than the nose, whereas a negative score would indicate more time spent, relatively, looking at the nose than the eyes. Based on the pupil differences we would expect that, after the first two seconds, gaze would spend significantly more time on the European face’s nose (a relatively darker region) and relatively less time on the African face’s eyes (a relatively brighter region). Instead, we expected this relative difference to be weaker or absent from onset or 0 ms until 2000 ms and, indeed, the mean difference score were both positive (African face: mean Dwell-time Difference = 9.00; *SD* = 25.5; European face: mean Dwell-time Difference = 1.04; *SD* = 25.4) and a paired t-tests, failed to reach significance level, t(23) = 2.03, p = 0.06. As expected, the dwell-time difference score for the following time window (from 2000 ms to 4000 ms) revealed a significant difference and in the expected direction, t(23) = 2.31, p = 0.03, since the score for the ‘African face’ score was positive and for the ‘European face’ was negative. That is, in the time window where pupils differed in size when attending to the either the African or European face, gaze spent relatively more time on the African eyes (mean Dwell-time Difference = 4.05; *SD* = 17.6) than on the European nose (mean Dwell-time Difference = -4.05; *SD* = 22.0).

We also analyzed separately the pupillary responses to the upside-down versions of the target faces with a similar ANOVA as for the upright faces. As expected, given that the faces’ ethnicity may be less clear with inversions, this analysis failed to reveal a significant main effect of Face Ethnicity, *F* (1, 78) = 3.79, *p* = 0.06, indicating that the differences in pupil diameters to the two faces were reduced in this condition (African face: mean pupil change = 0.085 mm; *SD* = 0.19; European face: mean pupil change = 0.55 mm; *SD* = 0.18). None of the other effects were significant (0.063 < *p* < 0.87). Given the absence of significant differences with the inverted faces, we did not further analyze the pupil evolutions over time as done with upright stimuli.

#### Implicit Attitude Test

A total of 17 Africans, 15 Asians, and 10 Europeans that participated in Experiment 2 were also able to complete the IAT online test on skin-tone right after the eye tracking session. On average, our participants scored in the range of “a slight to moderate preference for people with light skin than dark skin” in all three groups. However, a simple ANOVA analysis on the IAT scores and Participants’ ethnicity showed a significant difference, *F* (2, 39) = 5.01, *p* = 0.011. Post-hoc (Fisher’s PL*SD*) tests revealed that the Africans differed from the Asians (*p* = 0.004) by showing a relatively lower preference for light skin; the other across-groups comparisons were not significant. Most important, the implicit attitude scores did not predict pupillary responses according to correlation analyses between each participant’s IAT score and the individual’s pupillary changes to either the African (*F* = 0.3; *r*^*2*^ = 0.007) or the European face (*F* = 0.2; *r*^*2*^ = 0.005). This outcome with implicit attitude scores seems consistent with the previous findings by Levin and Banaji [1] using measures of explicit attitudes. However, the present results have to be interpreted with caution given the small number of participants from each ethnic group who completed the IAT.

### Discussion

By monitoring eye fixations over the faces of the classic demonstration of the face-race lightness illusion, we confirmed that observers did not distribute gaze equally over key parts, as the eyes and nose, of the African and European faces. Considering that these “central parts” of the faces do differ locally in luminance brightness (as visible in the image subtraction in Fig 3), there is good reason to believe that the observed overall oculomotor behavior for each face can differently stimulate the observers’ eyes leading to differing perceptions of luminance, each associated with one face. Put simply, despite the faces being equiluminant on average, they are perceived as different, because those regions that are fixated on the most are physically brighter or darker in each face.

Firestone and Scholl [27, 28] had argued that a bottom-up account of the FRL would be preferable to the original top-down, perceptual distortion, ‘prototype’ account and explicitly suggested that it might be sufficient to focus attention on dark regions of the African face and/or light regions of the European face in order to literally “see” one as darker than the other. However, the present findings failed to confirm this specific prediction about observers’ gaze behavior. On the contrary, we found the exact opposite pattern of overt attention. This finding seems puzzling and counterintuitive for the artifact-based account. At a first glance, the fact that observers looked more at bright parts of the subjectively darker face would seem to rule out a causal role of gaze for the experience of the illusion, since it the patterns of eye fixations seemed inconsistent with the perceived luminance in these stimuli. Nevertheless, we believe that the eye-tracking (and pupillometry) results may not at all be unrelated to the FRL bias and, on the contrary, help to understand how the illusory effect is generated.

As we saw in Fig 6, the eyes of the African face attracted more attention than those of the European face. Given that the eyes of the African face are relatively brighter (in the sclera) than those of the European face (see Fig 3), it would be expected that when directing gaze to the African face’s eyes it would lead to a reduced pupil size compared to when viewing the same region in the other face (see Fig 7). Moreover, the nose is fixated to relatively more in the European face than the African face and in the former it is also relatively darker, which is expected to result in a relatively larger pupil size when viewing the European face relative to the African face. Hence, it is reasonable to conclude that differential dwell time on the nose and eyes of each face may have been partially responsible for the observed changes in pupil diameter (i.e., we note that fixations on the eyes and nose combined accounted for an average 45% of all dwell time).

Interestingly, when the same face pairs were reversed in orientation on screen, so that the faces appeared upside down, it was now the European face’s nose that was the face part looked at the most (showing a 9% increase in dwell time compared to when the faces were shown upright as well as a decrease a 2–6% in attention to the eyes in both faces). The reduction in time spent on the eyes of the African face and the relative increase in dwell time on the European Face’s nose could consistently account for the null finding with pupil diameters when viewing the faces upside-down. Note that this is also in line with the independent findings of Experiment 1, showing that the inverted faces significantly decreased the FRL bias, repeatedly failing to reach significance for the African and Asian participants (see Table 1).

Crucially, our pupillary measurements revealed that the eye pupils’ diameters were relatively smaller when attending the African face than the European face. As seen in Fig 7, the pupil diameters tended to differ significantly after 2 secs from onset of the face pairs and these ocular differences were maintained during the following two seconds of attending to each face, most clearly for the Asian and European participants.

We note that many current models of social categorization assume that information about classes of individuals, like ethnicity, is encoded both 1) automatically and 2) rapidly [5]. An EEG study [64] concluded that changes in brain potentials related to “black” and “white” faces peaked with a mean latency around 120 milliseconds from onset of the visual stimulus. Considering that a cognitively-related pupil response may take a latency of at least 500 milliseconds to be expressed [48, 50], it seems reasonable to expect that pupillary changes in the present context will evolve slowly within the first second or two of exposure to the face stimuli.

One expectation about the pupillary changes is that, as gaze shifts from one face to another, the pupils adjust accordingly to the local differences in luminance. Interestingly, we found that the face part that was looked relatively more in the European face was the nose, which turns out to be relatively darker than that of the African face (Fig 6) and this pattern was more typical of the second half of the presentations (from 2000 ms to 4000 ms) than the initial 2 seconds, which is consistent with the findings of significantly different pupil size after 2000 ms. This is remarkable, given that looking at the eyes and nose, although the most overtly attended areas of interest, accounted for only about half of all fixation time, the rest of gaze’s dwell time being variously distributed over other areas and in a rather different manner across participants.

The above considerations about gaze location and pupil responses seem most relevant in the light of the ‘Predictive sampling’ account, which can make sense of both the counterintuitive gaze bias and the related pupillary changes. In fact, both of these can be seen as fundamental to the experience of the FRL. Specifically, we propose that the specific pattern of attentional sampling of local luminance in the faces causes systematic changes in retinal illuminance. For example, when the African face is attended, *relatively less light enters the pupil* than when attending the European face; consequently, the African face looks overall darker than the European face. This account hinges upon the following point: the total number of quanta per second that fall on the retina is directly proportional to the area of the pupil [65]; hence, the pupil diameter can affect the perceived light intensity of a visual scene. When the pupil widens, retinal illuminance increases [66] and the image of an object becomes more intense, while other properties, like the size of the retinal image (and features within) do not change. Indeed, if the pupil’s area would increase 50% from time_1_ to time_2_, then twice the amount of the quanta radiating from the image would be able to pass through the pupil and the image would be twice more intense. As an example, consider that the eye pupil reacts to a reduction in external illumination by dilating when a lamp light is dimmed; because such a response counteracts to some extent the reduction in light stimulation, there is always a difference between measured and perceived levels of illumination (e.g., a desk lamp output reduced to 10% of its maximum output is perceived to be as 32% or 3 times brighter than the measured light change; [65]). In general, according to research on the perception of ‘lightness’, this is a perceptual quality of surfaces or their reflected light that is inherently confounded with the illumination intensity, which can vary greatly over time and space in natural viewing [67, 68].

Most relevantly, even small changes in pupil diameter should result in noticeable changes in the perceived light intensity of the external stimuli. In fact, in the present experiment, the relative differences in diameter when observers looked at each of the two faces were rather small; that is, these differences reached maximums of 0.07 and 0.08 mm (between 2000 and 2400 ms from onset), corresponding to no more than a 2% difference in pupil diameters. Yet, this would correspond, according to the optical constraints between size of the aperture and amount of light passing through it, to a 3.3% change of the pupil’s area and of the quanta of light passing through the pupil. Interestingly, in the original psychophysical experiment by Levin and Banaji, their participants adjusted the brightness on screen of one of the two faces until the appeared to have the same luminance. Their morphs had a mean luminance adjusted to 141 units of pixel brightness (out of 0 to 255 in RGB/HSL pixel values) and their participants adjusted the black face to be on average 2.9 units higher than the (constant) white face, while the latter was adjusted 1.4 levels lower than the (constant) black face. Although the changes in pixels’ brightness were based on minimal differences in luminance (2% and 1% respectively), it would thus seem on the basis of their luminance-matching experiments that small step-changes of brightness at the input level, either by changing the amount of light entering the eye pupil, could make a significantly visible difference in the perception of these faces’ skin-tone luminance.

However, to understand fully the mechanisms that underlie the face-race lightness illusion, it seems crucial to consider not only a) the effects of gaze shifts over the sensory features within each of the two faces, but also b) the visual attention’s selection of the focus target. Specifically, one should expect sequential attentional shifts to obtain different levels of perceived luminance, each one associated with the object selected within the focus of attention. Several recent studies show that covertly attending a relatively darker or brighter stimulus than that at fixation elicits corresponding changes of the pupil (i.e. dilations for darker, constrictions for brighter), despite the luminance levels of the whole display remain constant [69–71]. We surmise that different ways of focusing attention could potentially impact pupil differences and it is possible that, from the first to the second half of the presentations in Experiment 2, observers became progressively more attentive at the eyes and nose while fixating them, which would enhance the impact of their differential luminance on the pupils, as indicated by the above studies.

We note that current accounts of the perception of lightness indicate that perceptual mechanisms for lightness do not simply compute overall constant luminance ratios but that the perception of lightness of a specific surface depends on how the visual image is initially parsed, e.g. by Gestalt rules and attention, and then by comparisons between weighted averages of lightness within a local framework relative to the global framework [67, 72, 73]. Moreover, ‘pupil perimetry’ studies indicate a central region of vision (about 5° large [74]) as most responsible for evoking pupil adjustments to light levels and the pupil’s sensitivity to light decreases rapidly with eccentricity [75]. Given that the faces’ size and distance on screen in the present study exceeded the extent of this central region, we surmise that the contribution to the pupillary response of the unattended face would be peripheral and reduced, if not negligible.

Our findings also hint at the possibility that African participants tended to show less of a pupillary difference than the other groups (se graph and functional t-test in Fig 7). We speculate that such a reduced difference may reflect a weaker FRL bias in the African group, which is not entirely inconsistent with the findings from Experiment 1, also showing that this group had several non-significant effects in the more challenging viewing conditions, where the faces were presented upside-down. However, in both experiments, the African samples were of smaller size than those of the other two groups and therefore the above suggestion should be taken with caution.

Finally, the participants’ implicit attitudes towards people of darker or lighter skin do not seem to explain the present pupillary responses, since scores on the IAT are not related to the pupil size changes. Also, all groups tended to show a preference for light skin. At any rate, if people were avoidant to the black face, one would have expected relatively larger pupillary dilations than to the white face, indicating increased arousal and emotional responses. Yet we observed the opposite. Also the following conclusion should be taken with caution, given the small number of completed IAT tests, but the IAT results in relation to the pupils are consistent with the idea that the face-race lightness illusion derives from categorical perceptual knowledge that is generally shared across ethnicities and independently of social attitudes towards out-group individuals.

## Experiment 3

If directing gaze on local regions of the faces and/or the related pupillary changes play a causal role in shaping the face-race lightness illusion, then a straightforward prediction is that constraining gaze to a single location and away from the faces should greatly reduce, if not eliminate, the FRL bias. In such a case, only the unchanging luminance at the fixation point would stimulate the eye and, therefore, any judgment about differences between the two faces should be based on their overall luminance. Hence, we presented the face pairs to each group of participants in either a ‘short’ (i.e., for 140 ms) or ‘long’ (i.e., for 2500 ms) presentation condition.

**Fig 8:**
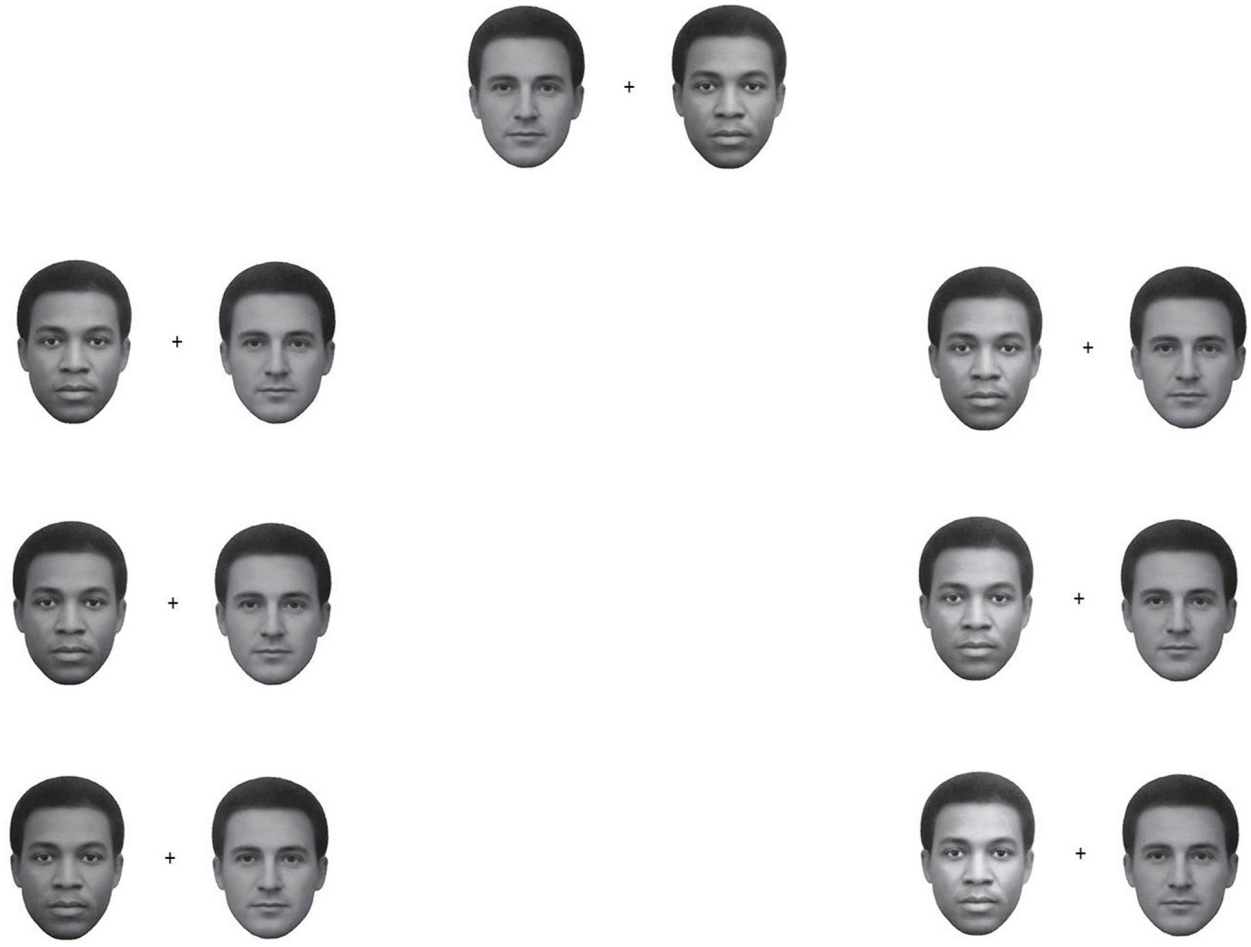
Stimuli used in Experiment 3. The top centered pair of faces corresponds to the typical FRL illusory stimuli, where the African and European faces are equiluminant (in this case: L_mean_ = 110.7, *SD* = 42). Below, the left column shows from the top Step -1, -2, and -3 in luminance of the African face (i.e., L_mean_ = 105, *SD* = 42; L_mean_ = 101, *SD* = 41; L_mean_ = 96, *SD* = 42, respectively) while the luminance of the European face is kept unchanged. The right column shows from the top Step +1, +2, and +3 in luminance also of the African face only (i.e., L_mean_ = 118, *SD* = 46; L_mean_ = 125, *SD* = 48; L_mean_ = 133, *SD* = 51, respectively). In the experiment, there were an equal number of trials in which the European face’s luminance was similarly manipulated while the African face was not and horizontally-flipped version were shown, so that each ethnicity would be equally likely to be either lighter/darker than the other or appearing to the left/right.

Our prediction is that for long presentations, where gaze is not constrained to a single fixation away from the faces, there should be a measurable FRL bias and judgments of the African face being darker than the European face should be more prevalent than the inverse. Moreover, this bias should clearly occur in trials in which the African face is slightly lighter in physical luminance than the European face, since a relatively lightened African face should be subjectively perceived of equal lightness to the unmodified European face [1]. We reduced in size the face pairs of the African and European morphs (see Fig 8 for an illustration of stimulus types), so that both whole faces were visible with good resolution while fixating at a central cross. In both conditions, the task was to indicate the lateral position of the ‘darker’ face.

Preliminarily, we verified that tachistoscopic (140 ms) and lateralized (away-from-fixation) presentations of the two faces were sufficient for observers to distinguish the ethnicity of the faces and regardless of the fact that in some trials the African face would lighter than the European and vice versa [3]. We tested 30 Norwegian students (19 females) at the University of Oslo (mean age = 25.9; SD = 5.1) which were asked to report on which side appeared an African face by pressing a key indicating a left or right position in relation to the fixation cross. Among the thirty participants, 28 had an accuracy rate above 75% correct and only two below this threshold (i.e., 71% and 68%) though both performed well above chance. Remarkably, 18 participants showed perfect accuracy (100% correct).

As revealed in Experiment 2 and illustrated in Fig 7 pupillary diameters evolved to be maximally different within 2.5 seconds from the onset of the face pairs, hence a ‘long’ presentation time should be sufficient for evoking the FRL illusion. We designed the experiment so that the face pairs had in some trials equal luminance, as for the stimuli in the previous two experiments, or one face was actually darker or brighter than the other. If a FRL illusory bias occurs, accuracy should be lower in trials where the African face is physically lighter and relatively greater when darker, since in the former case the illusory bias would counteract the increase in luminance whereas in the latter case it should increase the perception of a darker tone skin. Levin & Banaji [1] had performed a similar experiment with physically different luminance of the faces and a same-different task and ad lib stimuli presentations, although they described this “control” experiment only in a footnote. Their analyses of RTs revealed that the same-different judgments were slowed in trials with a relatively lightened African face.

For the present experiment, according to both the artifact and predictive sampling accounts, a ‘short’ or tachistoscopic presentation of the faces should effectively suppress the FRL bias; for the ‘prototype’ and ‘stereotype’ accounts, it should not be effective in changing the illusion, as long the observers are able to discern the ethnicity of the faces. Note, however, that the ‘artifact’ account explicitly suggested the possibility that covert attention could be a sufficient factor for modulating visual input, as long as there are salient luminance differences in the stimuli themselves consistent with a dark region to attend in the African face. Finally, the ‘predictive sampling’ account stresses the causal role of eye fixations for automatically triggering pupillary changes. Hence, this account makes the strong prediction that, by forcing fixation on a point external to the faces will lead to a constant pupillary size and erase the FRL bias.

### Methods

#### Participants

A group of 46 Norwegian students (22 females) at the University of Oslo (mean age = 26.8; *SD* = 5.5) performed the task with the ‘short’ presentations; another group of 41 Norwegian students (29 females) at the University of Oslo (mean age = 28.1; *SD* = 6.4) performed the task with the ‘long’ presentations.

#### Stimuli and procedure

The stimuli were face pairs of an African and a European man (see Fig 8 for an illustration of stimulus types). The stimuli used in the previous two experiments were reduced in size in *Adobe Photoshop* so that the whole faces were clearly visible while fixating at a central cross. Each ‘face’ image occupied a visual angle of 4x6° and they were positioned laterally to the fixation cross, so that the contour of the closest ear would be at a distance of 2° of visual angle. In ‘same’ trials, both faces had equal Mean Luminance = 113 ± .1 For the ‘different’ trials, we adjusted the average pixels’ brightness, either three steps up or down in HSL/RGB units, so that either the African or the European face could be physically brighter. The obtained stimuli had equal likelihood in showing a darker/lighter African face or European face and on either the left or right side of fixation.

Participants first followed a 4-point calibration routine. Following this, participants in the ‘short’ presentation group saw tachistoscopically a specific face pair, while fixating on a cross located between the two faces and at the center of the screen (Fig 8). This caused a small fixation cross to appear in the center of a blank grey screen. By use of the “AOI-trigger” feature in SMI Experiment Center, whenever participants fixated for 300 ms within a circular region around the fixation cross and invisible to the observers, the face pairs would automatically appear. This procedure guaranteed that, during the duration of the tachistoscopic presentation (140 ms), gaze would be on the fixation cross and none of the faces could be directly fixated with gaze before they disappeared from the screen. There were 38 trials in both the ‘short’ and ‘long’ presentation conditions, where 12 trials showed a physically darker African face and in another 12 a physically lighter African face, compared to the European face. In the remaining 14 trials, the faces were equiluminant The task was to report, by pressing one of three keys (B, N, M), on which side (B = left, M = right) they saw a relatively-darker face, or whether they appeared to have the same luminance (N = same).

### Results

We first examined accuracy scores, by computing percentage of trials in which a participant answered correctly that the African face was darker than the European face. We grouped trials according to whether the African (or the European) face was relatively ‘darker’ while the other face’s luminance was untouched, in three relative steps (see Fig 8); in an equal number of trials, the African (or the European) face could be ‘lighter’ in luminance, also in three relative steps.

**Fig 9:**
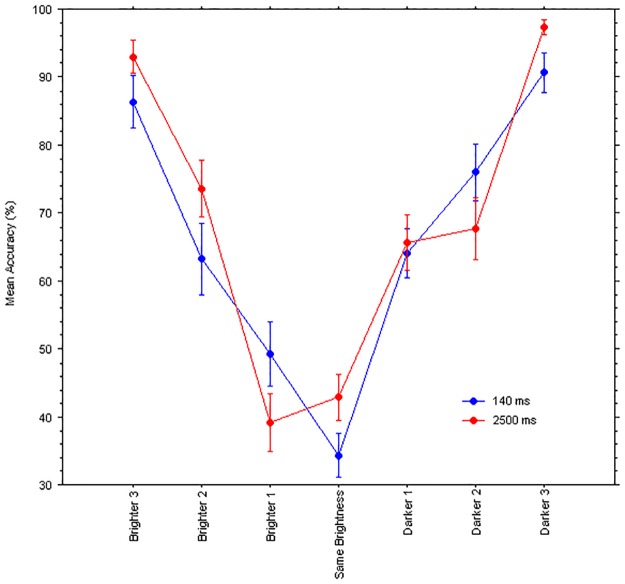
Experiment 3: Mean % accuracy in judging the African face as either brighter or darker or of same brightness as the European face. Bars represent standard errors.

A repeated-measures ANOVA, with Presentation Time (140 ms, 2500 ms) as a between-subjects factor and Luminance Steps (‘brighter’ +3, +2, +1, ‘same brightness’, ‘darker’ -1, -2, -3) as the within-subject factor, revealed again a significant effect of Luminance Steps, *F* (6, 510) = 63.42, *p* < 0.0001, η^2^_**p**_ = 0.4, no significant main effect of Presentation Time, *F* (1, 85) = 0.79, *p* = 0.4; but there was, importantly, a significant interactive effect between the two factors, *F* (6, 510) = 2.49, *p* = 0.022, η^2^_**p**_ = 0.3 (Lambda = 14.8; Power = 0.84). Fig 9 illustrates the results in both conditions and for the different luminance steps.

Pairwise t-tests revealed that the interaction was caused by the presence of a significant difference in ‘short’ presentations (140 ms) for ‘brighter’ 1 and ‘same brightness’, t(40) = 2.44, p = 0.019 (Cohen’s *d*: 0.44), whereas in ‘long’ presentations of 2500 ms) there was no significant difference between ‘brighter’ 1 and ‘same brightness’, t(45) = 0.62, p = 0.54 (Cohen’s *d*: 0.12).

As visible in Fig 9, accuracy in identifying which face was relatively darker was highest for the largest luminance difference (i.e., (‘brighter’ 3 and ‘darker’ 3) but performance decreased as the physical luminance became progressively similar (‘brighter’ 1 or 2; ‘darker’ 1 or 2) to being identical (‘same brightness’), where the percent of correct responses was no different from chance (i.e., 33%).

Because the prototype-based account would predict that a relatively lightened African face should be subjectively perceived of equal lightness to the unmodified European face (Levin & Banaji, 2006), we also looked at the frequency of incorrect ‘same’ responses in the conditions were the African face was made slightly ‘brighter’ or ‘darker’ (i.e., in the step -1 changes in luminance). In the ‘short’ condition (140 ms), a slightly lighter African face was judged to have the ‘same’ skin tone than the European face in a total of 43 trials, whereas in a total of 46 trials our observers judged it as actually darker. Remarkably, a slightly darker African face was also judged to have the ‘same’ skin tone than the European face in a total of 43 trials, whereas in a total of 23 trials our observers judged the latter to be darker. A Chi-square analysis of the ‘short’ presentations confirmed that these types of confusion errors did not differ significantly; Chi-square = 3.46, p = 0.06 (Fischer’s Exact p-value = 0.07). In addition, in trials where the two faces did not differ in luminance, i.e. in the ‘same brightness’ incorrect trials, our observers actually reported more often the European face (240 trials) than they reported the African face (215 trials) as darker.

In contrast, in the ‘long’ (2500 ms) presentation condition, the frequency of incorrect ‘same’ responses in the conditions where the African face was made slightly ‘brighter’ or ‘darker’ (i.e., in the step = ±1 change in luminance) showed an asymmetry in choices. That is, a slightly lighter African face was judged to have the ‘same’ skin tone than the European face in a total of 59 trials versus 12 trials judged as darker. A slightly darker African face was judged to have the ‘same’ skin tone than the European face in a total of 40 trials, whereas in a total of 19 trials our observers judged the latter to be darker. A Chi-square analysis confirmed that the distribution of these errors differed significantly; Chi-square = 4.15, p = 0.04. In ‘same brightness’ trials (total = 574 trials), where the two faces did not differ in luminance, our observers reported the African face to be darker in 212 trials and the European face in 209 trials.

In addition, for each individual participants we computed, using a calculator based on the formula by Macmillan & Creelman [76], the ‘hits’ (i.e. ‘same brightness’ responses to equiluminant face pairs), ‘false alarms’ (i.e. ‘same brightness’ responses to face pairs of different luminance), ‘correct rejections’ (i.e. ‘darker face’ responses to face pairs of different luminance) and ‘misses’ (i.e. ‘darker face’ responses to equiluminant face pairs). We then used the individuals’ *d’* and *C* values as dependent variables in independent-samples t-tests, with Time (140 ms, 2500 ms) as between-subjects factor. In signal detection theory [76], the *d’* value assesses how well two things can be distinguished and *d’* ranges from 0 (*no discrimination*) to infinity (*perfect discrimination*). If the Criterion (*C*) value is 0 then an observer’s response is not biased, showing no tendency for either response type (either ‘same’ or ‘different’ brightness); if C<0, there is a bias towards ‘same’ responses (with more ‘hits’, but more ‘false alarms’); if C>0 then there is a response bias towards ‘different’ responses. The mean *d’* in the 140 ms condition (mean *d’* = 1.104, *SD* = 0.73) did not differ significantly from the 2500 ms condition (mean *d’* = 0.958, *SD* = 0.69), t(83) = 0.96, p = 0.34. The *C* values were both positive, indicating a tendency towards ‘different’ responses in both conditions; however the bias was significantly larger in the 140 mw condition (mean *d’* = 1.073, *SD* = 0.63) than in the 2500 ms condition (mean *d’* = 0.721, *SD* = 0.58), t(83) = 2.65, p = 0.01.

We did not find significant differences in RTs between the ‘same brightness’ condition and the two conditions with slight changes in luminance; either for the ‘short’ presentations, *F* (2, 90) = 1.16, *p* = 0.31, or for the ‘long’ presentations, *F* (2, 84) = 0.13, *p* = 0.87.

Finally, we assessed the typical duration of an eye fixation on the faces during the ‘long’ presentations. All participants’ mean durations of eye fixations turned out to be longer than 200 ms, though a few participants showed average fixating times as long as 400–600 ms and the Mean Duration was 339.8 ms (*SD* = 88.2).

### Discussion

The goal of this experiment was to assess the presence of the FRL illusory bias when observers viewed face pairs where the luminance of one face was actually made physically ‘darker’ or ‘lighter’ than the other. One major aspect of this experiment was that, in the ‘short’ presentation condition, gaze was prevented from being directed at the faces. The findings clearly indicated that there was an absence of a bias in judging African faces darker than European faces with tachistoscopic (‘short) presentation times. This result is not consistent with the original ‘prototype-based’ account, since observers should a) be more likely to report the African face as darker than the European face as long as these are perceived as being of the respective ethnicities (an ability that is not affected by short presentation time away from fixation); and b) performance should be asymmetrical depending on whether the African face was made physically lighter or relatively darker; that is, the illusory bias should, on one hand, counteract relative increases in luminance of the African face and, on the other hand, yield a relatively darker impression when decreased. Hence, we conclude that the presence of the face-race lightness illusory bias depends on the direct stimulation of the eyes by the face stimuli.

In contrast and as expected, allowing gaze to move and sufficient time to allow pupil to change, resulted in the re-emergence of the FRL illusory bias. Our observers were indeed more likely to report in ‘long’ presentations that the African face as darker than the European face, as shown by the asymmetry in correct responses when the African face was actually darker than when it was actually brighter. In particular, the asymmetry was clear when the difference in luminance was slight (i.e., in ‘brighter 1’ ‘darker 1’ conditions; see Fig 9). The presence of an illusory bias was also revealed by a failure to indicate as brighter the African face when it was actually slightly brighter, which seems consistent with the idea that the increase in physical luminance of the African face was counteracted by the illusory bias. Indeed, according to the ‘predictive sampling’ account, the likelihood of reporting the African face as darker than the European should be symmetrically affected by increases or decreases of relative luminance between the two faces.

Because the major difference between the two conditions was that gaze was prevented from being directed at the faces in the former and free in the latter, we conclude that the two sets of results converge on the conclusion that the face-race lightness illusion bias depends on directing gaze onto the faces. Interestingly, the mean duration of fixations during the ‘long’ presentations was of about 400 ms, which would seem more than sufficient for pupillary adjustments to take place and to affect the level of retinal illuminance while simultaneously attending overtly to one particular face.

One should also note that in the present forced-choice task, participants had knowledge that sometimes the faces would have the same luminance. We surmise that this expectation might have influenced them to opt for a guessing strategy whenever the faces looked sufficiently similar to one another in luminance. Hence, in both the condition in which they were actually identical and the one where the African face had slightly lighter luminance (‘brighter’ 1 in Fig 9), accuracy rates did not differ from chance level. There was however a significant increase in ‘same’ luminance choices in incorrect trials in the latter condition, which is also consistent with a FRL bias. A signal-detection analysis revealed that discrimination sensitivity was comparable in the ‘short’ and ‘long’ presentations. However, the *C* values indicated a larger bias to respond that the faces were ‘different’ in luminance in the 140 ms condition than in the 2500 ms condition, which would seem consistent with the idea that when the FRL illusion is present, as in the latter condition, observers are less biased to report one face as different than the other (especially in the condition in which the African face is actually slightly brighter; see Fig 9).

Finally, although Levin & Banaji [1] had found, in a same-different task, slower responses in the condition with a relatively lightened African face, we failed to observe any change in speed of response. In fact, if taking RTs as a measure of processing efficiency [77, 78], it would seem that efficiency was not sensibly affected across the various conditions and experiments, despite the lower visual resolution of the stimuli in Experiment 3.

## General discussion

The face race lightness illusion appears to affect a majority of individuals, independently of their ethnicity, though not everyone shows the bias consistently. The novel finding of the present study is that FRL illusory effect appears to be dependent on the pattern of eye fixations. Counterintuitively, gaze patterns are not consistent with fixating relatively darker face regions of the African face and/or relatively lighter regions of the European face as suggested as a possibility by an ‘artifact’ account of the bias. In fact, in the present findings, the opposite pattern is observed. Far from suggesting that oculomotor behavior is irrelevant to the illusory experience, the following empirical evidence collected here confirms that: 1) constraining gaze away from the faces erases the bias; 2) pupil diameters are concomitantly and systematically related to gaze being focused on each stimulus.

Specifically, we suggest that the pupillary changes affect the level of retinal illuminance associated with the attended face, resulting in a perception of differing luminance that is consistent with learned facial prototypes or perceptual priors. Indeed, in Experiment 2, we observed significant differences in pupillary responses to each face, most clearly in the Asian and European groups. In Experiment 3, with tachistoscopic presentations, European faces were just as likely to be judged as darker than the African faces when the face luminance differed between the two. Moreover, responses were not significantly biased towards African faces being seen as darker at any of the tested levels of luminance differences and incorrect ‘same’ responses did not differ in their frequency when the African or the European faces were made slightly darker or brighter respectively and the African face was not reported more frequently as ‘darker’ than European face.

All in all, the present constellation of findings seems best explained by a ‘predictive sampling’ account where the visual system attempts to minimize the difference between perceptual categorical knowledge and visual input. In other words, the phenomenology of the face-race lightness illusion may derive from the initial activation of learned, ethnic-specific, “perceptual templates” or face prototypes evoked by differences in the shape features of the faces. We surmise that by fixating prevalently on the brighter areas of the African faces, the resulting changes in pupil diameter would gradually modulate retinal illuminance [58] and therefore the intensity of light input associated when attending to a face. In other words, changes in pupil size would underwrite the illusion by countering surprising deviations or noise from expected values, reducing the mismatch or “dissonance” between visual input and prior knowledge. Interestingly, other research indicates that the relative size of the pupils can affect several aspect of how we interpret light information, including the color features of the so-called *#thedress* “illusion”, which are preferentially seen as “black and blue” by individuals with large pupils, whereas it looks “white and gold” by those with small pupils [79].

In general, the presence of an illusion is a tell-tale of the assumptions that the mind relies upon when ‘inferring’ perceptual information, especially when the sensory data are ambiguous [80]. In the presence of a mismatch between prediction and sensory evidence, the visual system is challenged to make perceptual inferences on stimuli and these inferences can often result in dramatic departures of the visual experience from the physical input [81]. In other words, perception settles on a specific interpretation of the ambiguous sensory input on the basis of a pragmatic principle of optimizing behavioral responses, more than on a principle of veridicality [82, 83]. In the domain of pupillary responses to optical illusions of luminance [84], it has been proposed that illusions of strong brightness or “glare” [85–87] can powerfully engage the brain/mind to expect forthcoming probable changes in light exposure [81], despite the locus of the subjectively intense light source has always the same physical luminance of the background. Moreover, simply imagining objects seen in bright light [56] or a shift of covert attention from a darker to a brighter stimulus [57, 69, 70] can both result in constrictions of the pupil.

According to Levin and Banaji [1], the perceived lightness of the faces result from a “distortion” of the conscious percept imposed by the “relatively abstract expectations about the relative reflectance of objects”. Such a distortion could be re-phrased as a case of a brightness *filling-in* process [88, 89] or world-knowledge based color “recalibration” [90] that causes each stimulus to be “cortically” re-represented–after correcting the output of the initial sweep of processing—with a relatively lighter or darker tone. However, based on the present results, we can exclude that the pupils automatically adjusted to the luminance or brightness features coded in the neural representation of the inferred percept. That is, if each face’s skin tone were “filled-in” according to the expected differences, this should in turn elicit adjustments of the pupil towards a smaller/wider aperture in relation to the brightness features represented in the brain state (as shown by imagery studies; [56]). However, we found the opposite relationship between pupil size and FRL bias, suggesting that both a “reactive” account for pupil response and a “corrective” account for the percept may be incorrect.

To conclude, no previous study has elucidated the process behind this particular illusion in a mechanistic way. In our opinion, an appeal to top-down processes is plausible but it remains vague and does not specify where within the hierarchy of perceptual processing the bias is generated and sustained. We propose that such a “top-down” influence could bias attentive, oculomotor-based, mechanisms so that the brain attempts different sampling modes of the input before settling for an interpretation of sensory information that minimizes the difference from the expected stimuli. In other words, predictions based on typical skin tones become self-fulfilling prophecies, by treating input as noise and settling perceptual inferences in favor of the learned statistical regularities in the external input. Consistent with this “hypothesis testing” view of perception that is common to many computational and psychological accounts of perception and its illusions [91, 92], including the recent ‘predictive coding’ account [83, 92–95], the control of gaze can play a key role in disambiguating the stimulus [96, 97]. In particular, Ulric Neisser [98] had envisioned perception as a cycle where an internalized schema leads to a readiness to perceive certain information that in turn guide the exploration of the environment which results in how an object is perceived [99]. In the present case, differences in the anticipatory representations influence the sampling of visual input from the two facial patterns which in turn differentially stimulate the pupils and generate separate perceptual experiences of brightness. Hence, the evidence presented in this study suggests that the underlying mechanism responsible for this specific illusion is already at the early level of sensory acquisition and attentional control.

## Appendix A

In Experiment 1, the largest group of respondents was located within Europe (N = 495): Albania (N = 2), Austria (N = 11), Belgium (N = 10), Czech Republic (N = 4), Denmark (N = 1), Estonia (N = 2), Finland (N = 1), France (N = 8), Germany (N = 6), Greece (N = 2), Hungary (N = 11), Ireland (N = 2), Italy (N = 97), Latvia (N = 2), Lithuania (N = 2), Malta (N = 1), Netherlands (N = 13), Norway (N = 84), Poland (N = 111), Portugal (N = 12), Serbia (N = 1), Slovakia (N = 2), Slovenia (N = 2), Spain (N = 6), Sweden (N = 8), Switzerland (N = 2), United Kingdom (N = 54). The second largest group was respondents was located within Asia (N = 246): Afghanistan (N = 1), Azerbaijan (N = 1), China (N = 1), Hong Kong (N = 11), India (N = 63), Israel (N = 1), Japan (N = 154), Jordan (N = 2), South Korea (N = 3), Taiwan (N = 4), Turkey (N = 2). There were also participants responding from countries in Africa (N = 68): Algeria (N = 2), Cameroon (N = 1), Guinea (N = 1), Kenya (N = 22), Nigeria (N = 38), Tanzania (N = 2), Uganda (N = 1), Zimbabwe (N = 1). The smaller groups were located in North America (N = 49): Canada (N = 5), United States (N = 44). Finally, two respondents were located in Australia, and two more in Central America (Mexico) and South America (Venezuela).

The combined responses to the Ethnicity and country of residence items clarified that many participants who had selected ‘Other’ ethnicity (N = 83) instead of ‘African’ (N = 76), ‘Asian’ (N = 246) or ‘European’ (N = 417) were either African American or of mixed ethnicities. However, several respondents also specified that they were either “white”, “Polish”, “Norwegian”, etc. For example, all of the respondents from India (N = 63) selected ‘Asian’ for their ethnicity. Indeed, nationality is often used synonymously with ethnicity in several contexts since a person’s ethnic identity is subjected to a variety of factors, like shared cultural heritage, ancestry, history, homeland, language or dialect, symbolic systems such as religion, mythology and ritual, cuisine, and the arts.

We excluded from the analyses of Experiment 1, participants who had chosen ‘Other’ from the analyses shown in Table 1, but we included in this restricted analysis three ethnic groups, here labeled ‘African’, ‘Asian’, or ‘European’, by selecting as African only participants who specified not only their ethnicity as ‘African’ but also their country as Cameroon, Guinea, Kenya, Nigeria, Tanzania, Uganda or Zimbabwe. Similarly, we selected as ‘Asian’ all the respondents that not only selected this ethnicity but who also declared to be from Far East countries (i.e., China, Hong Kong, Japan, South Korea, and Taiwan). The European group included respondents from Europe (e.g., Norway, Poland) or North America (e.g., Canada), unless they had further specified their ethnicity as different from ‘European’ (e.g., being an ‘Asian’ or ‘African’ residing in America or Europe).
